# Iron(II) Mediated Supramolecular Architectures with Schiff Bases and Their Spin-Crossover Properties

**DOI:** 10.3390/molecules28031012

**Published:** 2023-01-19

**Authors:** Dawit Tesfaye, Wolfgang Linert, Mamo Gebrezgiabher, Yosef Bayeh, Fikre Elemo, Taju Sani, Nandakumar Kalarikkal, Madhu Thomas

**Affiliations:** 1 Department of Industrial Chemistry, College of Applied Sciences, Addis Ababa Science and Technology University, Addis Ababa P.O. Box 16417, Ethiopia; 2 Nanotechnology Center of Excellence, Addis Ababa Science and Technology University, Addis Ababa P.O. Box 16417, Ethiopia; 3 Institute of Applied Synthetic Chemistry, Vienna University of Technology, Getreidemarkt 9/163-AC, 1060 Vienna, Austria; 4 School of Pure and Applied Physics, Mahatma Gandhi University, Kottayam 686560, Kerala, India

**Keywords:** iron(II), supramolecular, Schiff base, spin-crossover

## Abstract

Supramolecular architectures, which are formed through the combination of inorganic metal cations and organic ligands by self-assembly, are one of the techniques in modern chemical science. This kind of multi-nuclear system in various dimensionalities can be implemented in various applications such as sensing, storage/cargo, display and molecular switching. Iron(II) mediated spin-crossover (SCO) supramolecular architectures with Schiff bases have attracted the attention of many investigators due to their structural novelty as well as their potential application possibilities. In this paper, we review a number of supramolecular SCO architectures of iron(II) with Schiff base ligands exhibiting varying geometrical possibilities. The structural and SCO behavior of these complexes are also discussed in detail.

## 1. Introduction

**Figure 1:**
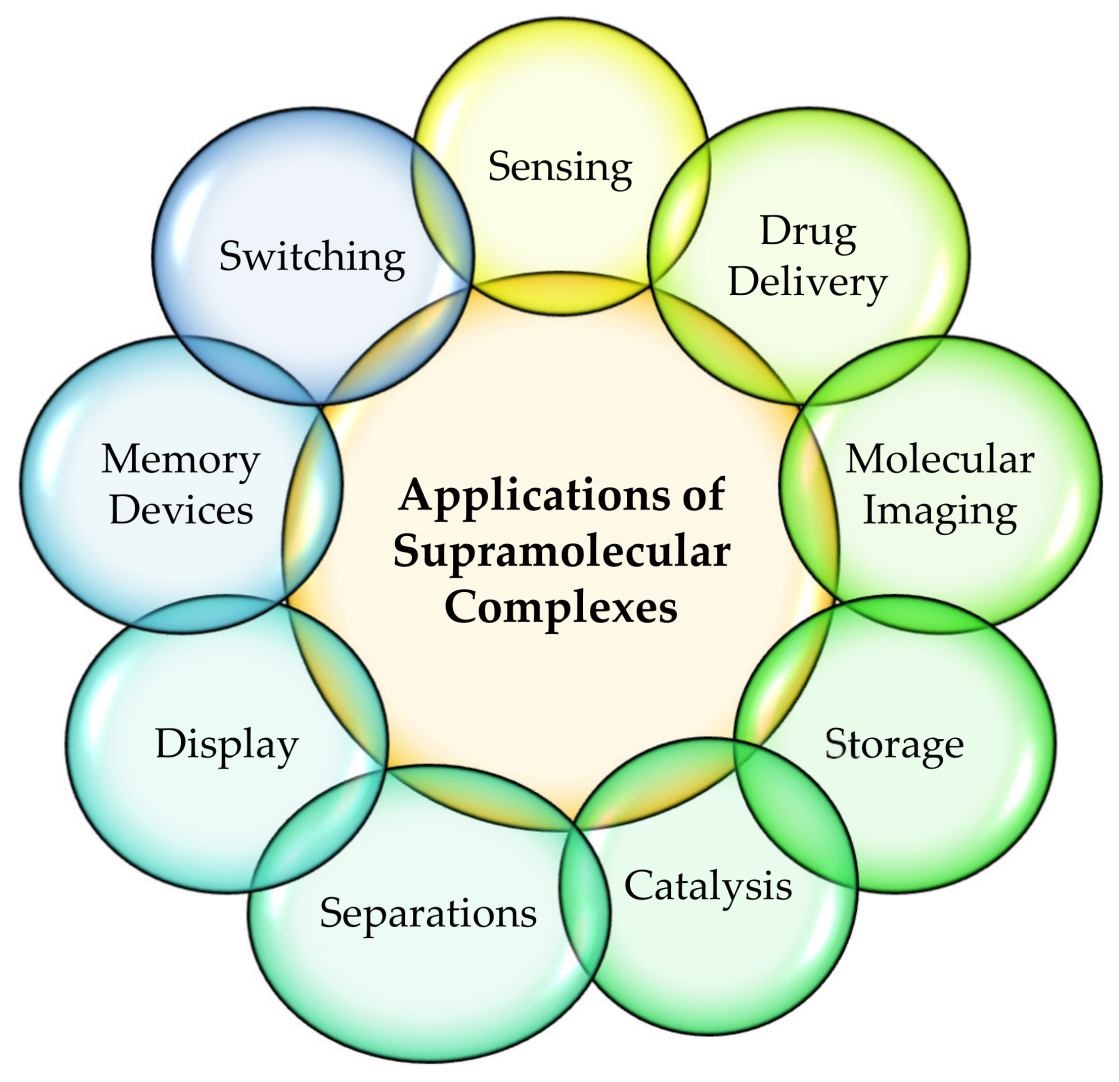
Applications of supramolecular complexes in host-guest chemistry.

Supramolecular coordination complexes (SCC), metal-organic framework (MOF), or discrete metal-organic assemblies (DMOA) are some of the pioneers in modern chemical structures that break the boundaries between inorganic metal cations and organic ligands [1,2]. The architecture of these supramolecular compounds shows great potential to create new variable molecules and materials that have diverse applications (Figure 1) [3,4,5,6]. In metal-mediated supramolecular architectures, the self-assembled structures can be arrived at through non-covalent interactions such as hydrogen bonds [7,8], halogen bonds [9,10], π-π interactions [11,12] and coordination bonds [13,14]. The chemical and physical interactions of different strengths and varying degrees of geometrical preferences are important to design and create different structures [15]. 

Metal-mediated architecture in supramolecular chemistry is one of the combinations of metal ions and organic ligands by self-assembly through coordination bonds [16]. The strength of the coordinate bond between metal ions and organic ligands shows both steady and dynamic character in sophisticated environments since its strength lies between weak noncovalent and covalent interactions [17]. In supramolecular chemistry, metal ions and organic linkers can be coordinated by Lewis acid/base interactions [18]. The basic aspects in metal-mediated supramolecular architectures during self-assembly are their adjustability to be designed as needed and their rational combinations of metal–ligand coordination for specific purposes, which can make them unique for many applications [19,20].

In the field of supramolecular chemistry, metal ions are being extensively used because of their inimitable capacity to organize molecules or atoms in a unique geometry [21,22]. Coordinating metal ions to organic linkers in supramolecular compounds has a great role in the adjustment of self-assembly and to modify the morphologies of the resulting supramolecular architectures. The metal ions, along with the binding ligands, determine the properties of the metal-coordinated supramolecular array because various metal ions differ in size, binding affinity, coordination geometry, coordination number and charge density [23,24].

The present review is concerned with self-assembled iron(II) systems showing SCO behavior with acyclic Schiff base ligands.

## 2. Schiff Base Ligand System in Supramolecular Architectures

**Scheme 1:**
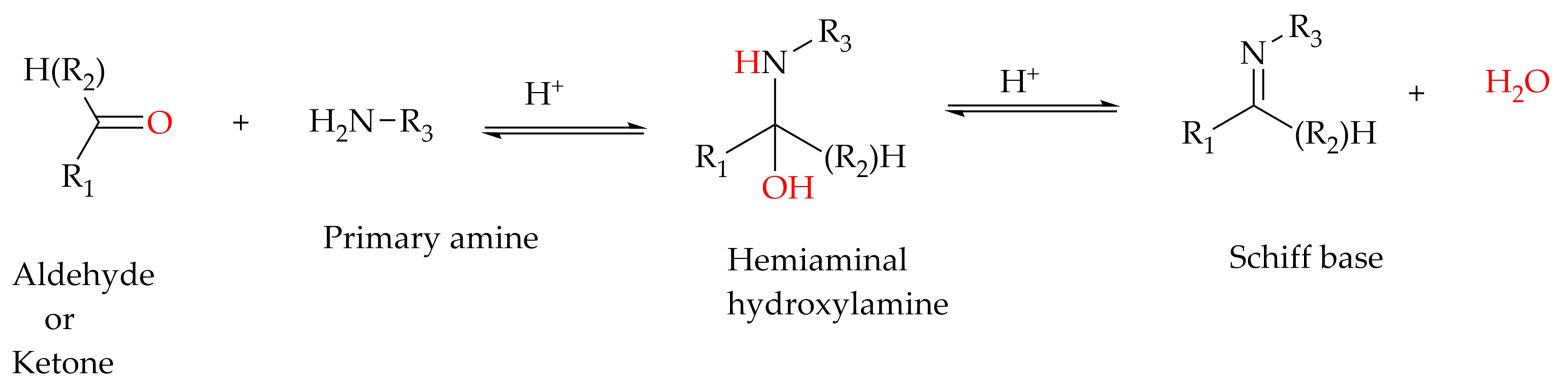
General schematic synthesis of Schiff base ligands. Reproduced from [25]; published by Wiley, 1864.

Schiff bases, named after Hugo Schiff [25], are organic ligands that are synthesized from primary amines and carbonyl compounds (aldehydes/ketones) and known as imine or azomethine. Structurally, a Schiff base is an analogue of a ketone or aldehyde in which the carbonyl group (-C=O) is converted by a primary amine into an imine or azomethine (-HC=N-) functionality [26] (Scheme 1). Schiff bases are well-known ligands in constructing supramolecular architecture and other complexes through coordination bond, hydrogen bond, halogen bond or π-π interactions [27]. Because of the simple reaction conditions to promote the synthesis of a wide variety of Schiff base ligands with different chelating abilities, flexibility or chirality, they are widely used to design and construct supramolecular architecture for specific purposes [27]. Moreover, depending on the shape of the formyl and amine precursors and/or the metal-ion template used, the supramolecular architecture of the macrocyclic molecules of helicate/meso-helicate/spiral, grid/square cage and cube-containing imine groups can be formed through cyclization reactions as well. Schiff bases are classical organic ligands for the metal ions of p, d, and f blocks, which have significantly contributed to the development of coordination chemistry on both basic and application aspects [28].

## 3. Magnetic Properties of Iron(II) Spin-Crossover Schiff Base Supramolecular Architectures

SCO supramolecular complexes are a fascinating class of materials revealing molecular bi-stability with their varying potential applications [29]. The molecular bi-stability is due to the ability of electronic state switching between high spin (HS) and low spin (LS) when external stimuli such as change in temperature, pressure, light irradiation or guest presence/absence occurs [30]. 

**Figure 2:**
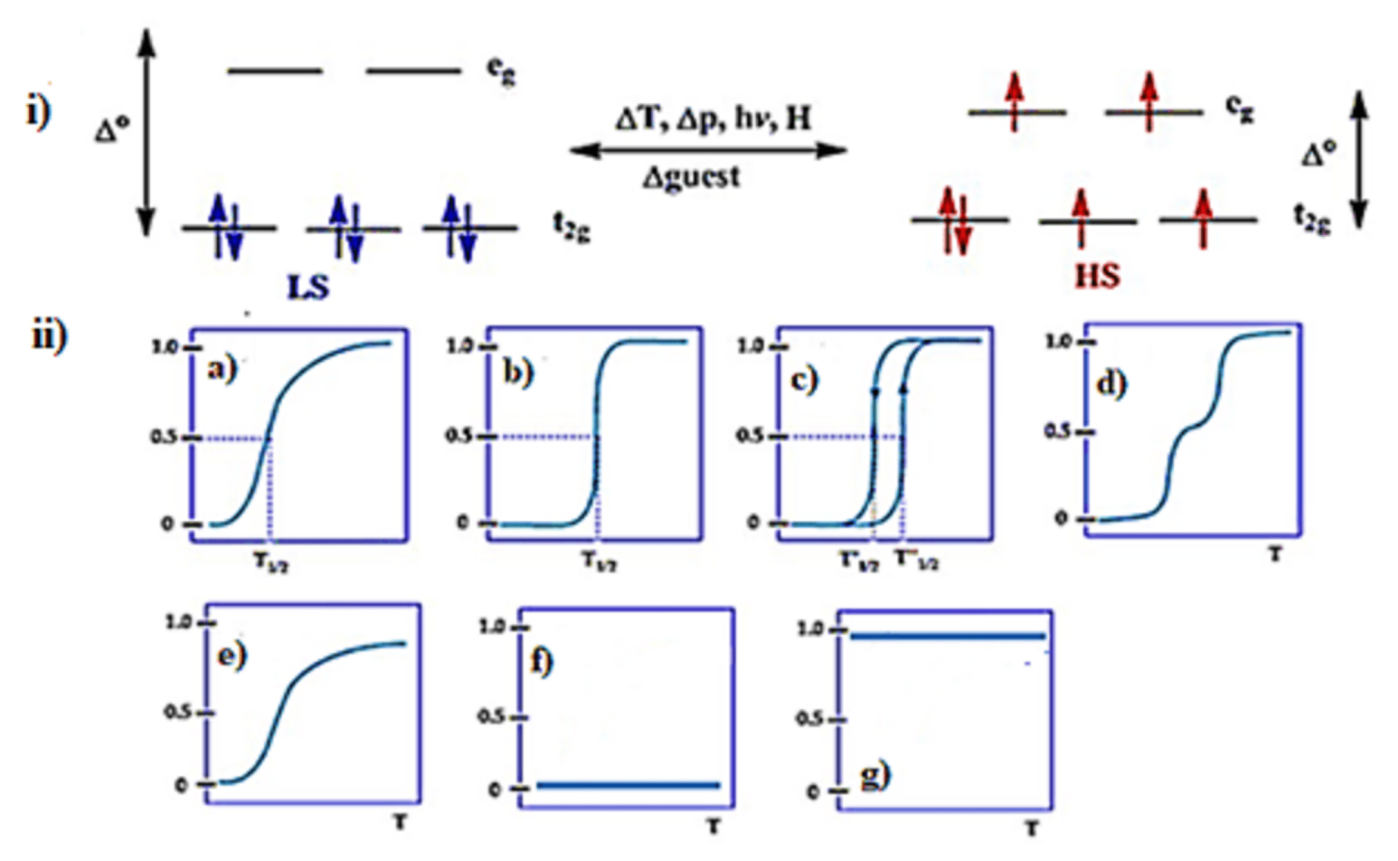
(**i**) The d orbital splitting diagram for an octahedral iron(II) ion. (**ii**) Various types of SCO behavior: (**a**) gradual (**b**) abrupt (**c**) with hysteresis (**d**) two-step transition (plateau) (**e**) incomplete (**f**) low spin and (**g**) high spin. Reproduced with modification with permission from [32]; Published by Royal Society of Chemistry, 2019.

SCO can be observable for octahedral 3d transition metal (3d^4^–3d^7^) ions. There are two ways of electron distributions in the t_2_g and e_g_ orbitals of octahedral iron(II) complexes. Depending on the nature of the ligand field splitting energy and pairing energy (P), electrons can be populated, as shown in (Figure 2i). When a strong ligand field is applied, there will be large Δo, and electrons will remain in the lower energy state of the t_2_g set and pair up, and it is a LS state (Δo > P), thus diamagnetic complexes can be observed. On the other hand, if weak field ligands are applied, Δo is small due to a very small energy difference between the t_2_g and eg sets and the maximum number of unpaired electrons, i.e., the HS state occur (Δo < P) [31].

When Δo is of a comparable magnitude to P, a possibility of inducing a switching between the HS and LS states arises (i.e., SCO) by perturbing the system through the application of an external stimuli (Figure 2i), by pressure, magnetic or electric field, light irradiation, and the presence/ absence of guest molecules. However, the most common perturbation is a change in temperature, due in large part to facile application and measurement [33].

The SCO/spin switching/spin state in complexes are classified as gradual, abrupt, hysteretic, stepwise, incomplete, low-spin and high-spin, as shown in (Figure 2ii). From spin switching in supramolecular complexes, hysteresis is the more focused one because of the cooperative first-order spin-state switching which is termed bi-stable. The supramolecular complexes in hysteresis spin switching can exist in two stable electronic states. The bi-stability of SCO supramolecular complexes, which shows a relatively large thermal hysteresis (ΔT ≥ 45 K) centered around room temperature (RT), is desired for various applications—especially for obtaining molecule-based switching and memory devices [34,35].

SCOs at the metal centers of supramolecular architectures cause the bond between metal and ligand to contract or expand by 5–10% in length [36]. This causes a change in the overall size and shape that leads to local distortions of the architecture. The distortions occurring in particular areas can trouble the lattice pressure at the nearest neighbor-switching sites that help transition propagating through the molecules. The efficiency of this propagation in the architecture determines the cooperativity of the metal centers in spin switching that exhibits thermal hysteresis [36,37,38]. Hence, the designing of hysteretic SCO materials for specific application requires controlling of the interactions in metal ion switching sites in the architecture. Materials that exhibit large SCO hysteresis are mostly molecular complexes [39,40,41], and many of the compounds are iron(II) complexes of N-donor ligands such as Schiff bases that are suited to cooperative spin transitions as they show large structural differences between HS and LS states [42,43,44].

SCO systems, from a materials point of view, are verified as a potential molecular switch component in memory storage devices. Spin switching research is targeted towards the direction of molecular switch and memory applications that lead towards SCO materials that have complete, abrupt, hysteretic and switchable characters regarding RT. Above all, complete transitions are preferred as they are easier in the detection of distinct fully HS and LS spin-switch states [45]. Abrupt transitions allow for a large output (color) for small inputs (small change in temperature). Hysteresis provides a thermal history of the complexes by the detection of temperature in the hysteresis loop; the system is bi-stable, and hence, there is a binary potential storage. Supramolecular array ligands that are designed for the purposes of giving exact Δo in the SCO, and also which facilitate cooperativity within or between the metal centers of complexes, are the main goal of many SCO chemists in getting abrupt and /or hysteretic spin transitions [46]. 

**Figure 3:**
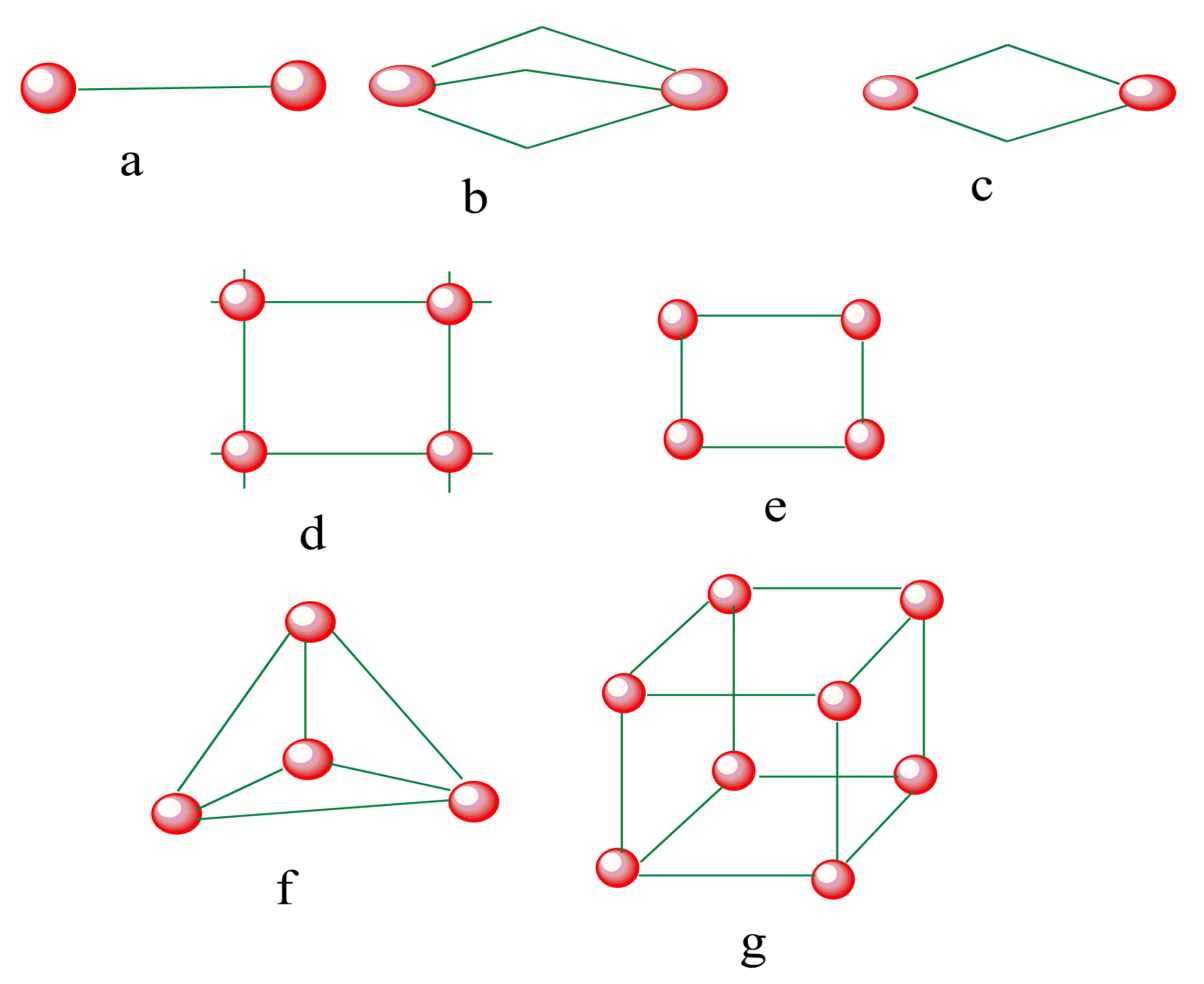
SCO-active complexes of di-nuclear (**a**–**c**); grid (**d**), square (**e**), tetrahedral cage (**f**), octahedral cube (**g**). Green = di- to polydentate of N-donor and O-donor Schiff base ligands. Red = iron (II) metal centers.

Mononuclear iron(II) SCO complexes that are constructed by different kinds of coordination environments have been studied extensively, while di-nuclear to poly-nuclear structures are rare [32,47,48]. Figure 3 shows the different geometric possibilities of the SCO iron(II) supramolecular complexes of di-nuclear (spiral, helicate mesocate), tetra-nuclear (grid, square, tetrahedral) and octa-nuclear (cube). Di-nuclear and poly-nuclear have advantage over monomeric complexes because of multiple metal centers that can be covalently bridged in enhancing cooperativity in SCO systems [49] with a designed and controlled synthetic protocol [50,51]. 

Time-temperature integration on SCO compounds with thermal hysteresis has been described by Cavallini et al. [52]. It is inferred from their review that in devices, the temperature band controllers (TBCs) are capable for monitoring the temperature changes depending on the spin states.

Also, in a recent review [53] Cavallini et al., described the integration of SCO compounds as thin films on surfaces for technological application, proving the advancement made on SCO compounds in thin film growth and the self-assembly of the materials on surfaces.

### 3.1. Iron(II) SCO Compounds with Spiral Architectures

Spiral architectures of iron(II) are an interesting area to investigate due to the fascinating structure and cooperative SCO behavior, as metal centers in those will lead up to three different spin states: LS-LS, LS-HS and HS-HS [54,55,56]. These spin states can be related to cooperativity. Negative cooperativity can lead to a two-step spin transition, as the first SCO can stabilize the LS-HS mixed spin state in (Figure 2d) and the second SCO takes place at different temperatures. Spiral iron(II) SCO architectures are interesting due to the fact that they have three-dimensional structures. As positive cooperativity occurs between metal centers, the SCO behaviors have been found in different forms compared as mononuclear analogues [32,54,55,56]. Positive cooperativity can lead to one-step spin switching of both metal centers (i.e., [HS-HS] to [LS-LS] or vice versa) in which a sharp, abrupt and/or hysteretic loop SCO can be attained (Figure 2b,c) [57,58,59,60].

Recently, a helicate-like di-nuclear macrocyclic Schiff base iron(II) complex was reported by Zhang et al. [61] showing an antiferromagnetic interaction between the metal centers. In the literature, there have been many iron(II) macrocyclic Schiff base complexes reported [61,62,63,64,65,66] which were not formed by self-assembly and did not show any SCO properties. 

**Chart 1:**
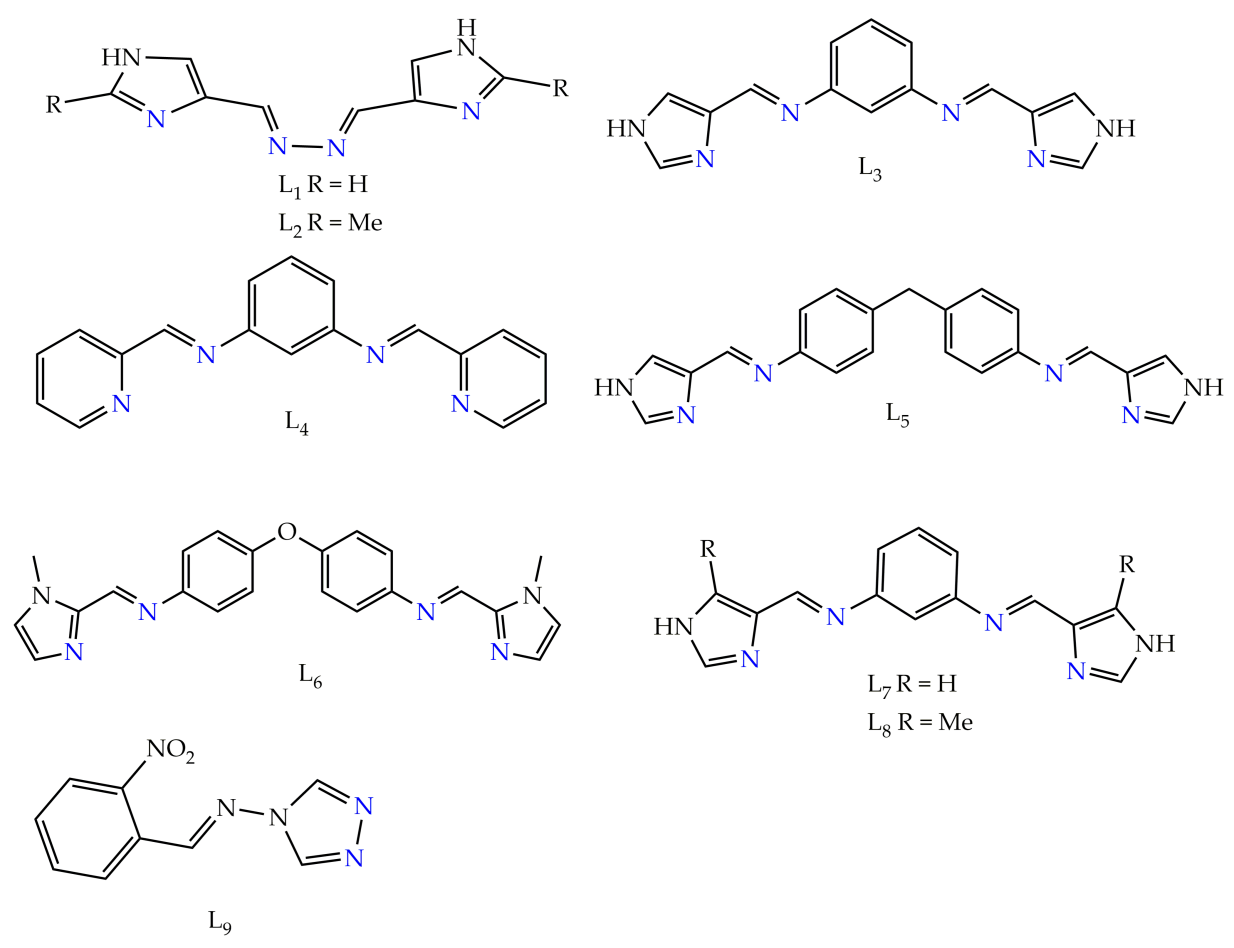
Ligands known to form SCO-active iron(II) dinuclear supramolecular architectures. Blue = binding pocket of the ligand to central metal.

**Figure 4:**
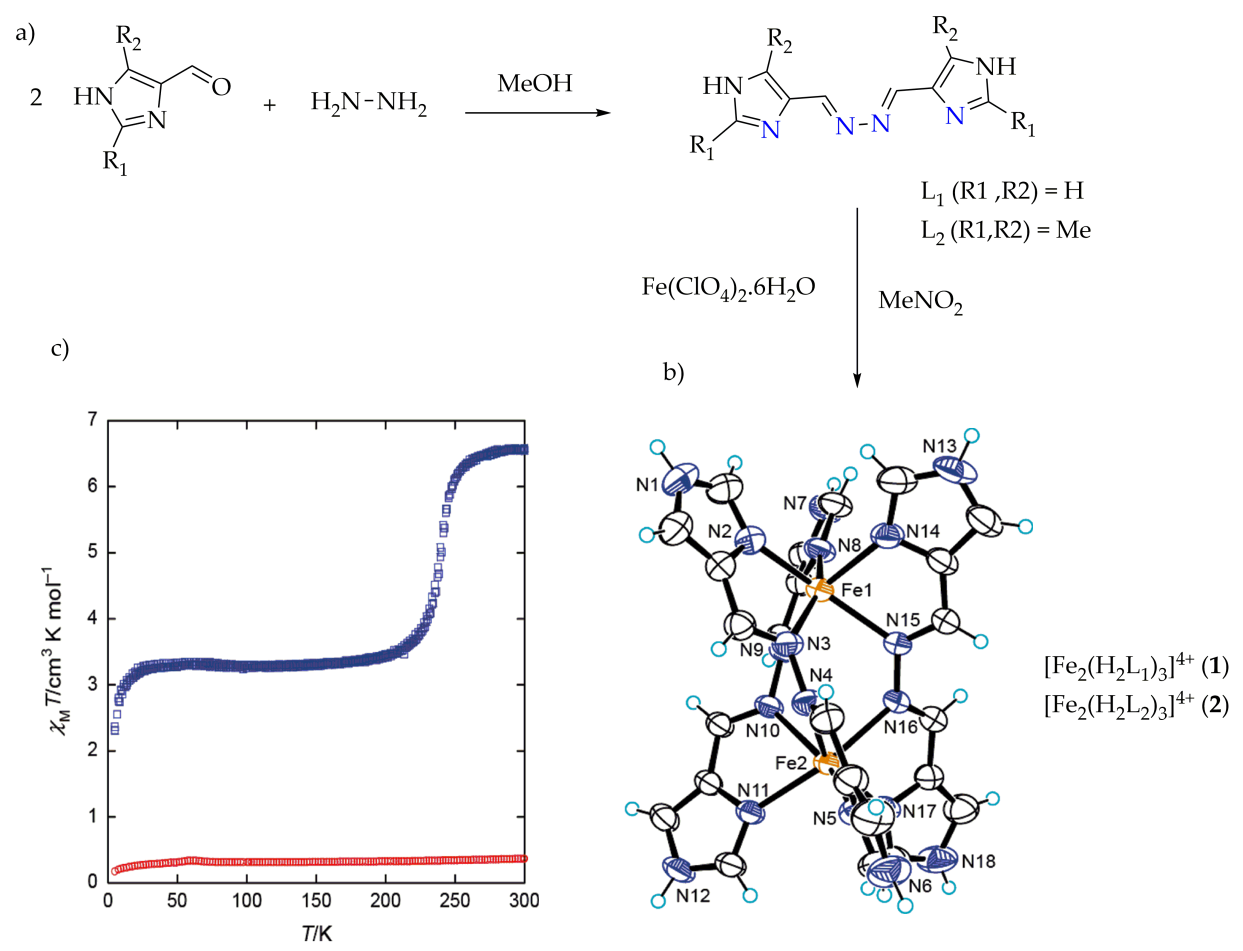
(**a**) L_1_ and L_2_ used to synthesize spiral iron(II) complexes. (**b**) X-ray molecular structure of the cation of complexes (**1**) and (**2**). Color code: orange = Fe; blue = N; black = C; light blue = H. (**c**) Magnetic behaviors of complex (**1**), (blue) and (**2**) (red) in the form of a χ_M_T vs. T plot. Reproduced with permission from [59]; Published by American Chemical Society, 2009.

Kojima et al., reported on the SCO of spiral iron(II) complexes of L_1_ and L_2_ of (Chart 1) [57,59]. The di-nuclear spiral iron(II) complexes [Fe_2_(H_2_L_1_)_3_]^4+^ (**1**), [Fe_2_(H_2_L_2_)_3_]^4+^ (**2**) were synthesized by the reaction of respective ligands (Figure 4a) and Fe(ClO_4_)_2_·6H_2_O in nitromethane in a 3:2 molar ratio (Figure 4b). The magnetic behaviors of complexes (**1**) and (**2**) are shown in (Figure 4c). The complex from L_2_ stayed in the LS state over the entire temperature range, and this result is in accordance with the strong ligand field strength of H_2_L_2_ caused by the presence of electron-donating methyl groups. The complex from L_1_ exhibited an abrupt SCO behavior at about 240 K. 

**Figure 5:**
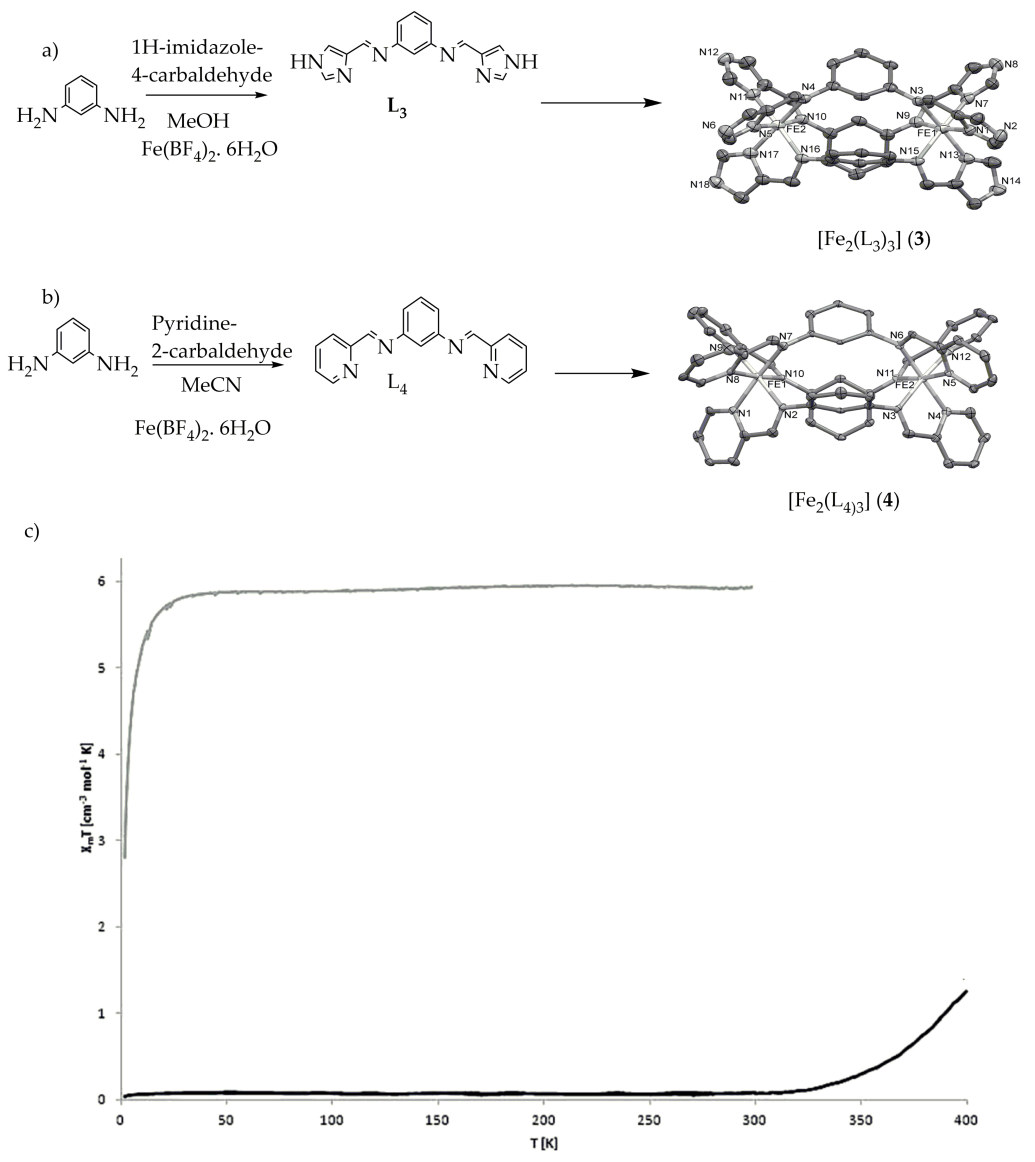
(**a**) [Fe_2_(L_3_)_3_] (**3**) [L_3_ = *N*,*N*′-bis(1*H*-imidazol-4-ylmethylene)benzene-1,3-diamine] (**b**) [Fe_2_(L_4_)_3_] (**4**) [L_4_ = *N*,*N*′-bis(pyridin-2-ylmethylene)benzene-1,3-diamine] (**c**) Magnetic behavior of complexes (**3**) blue line and (**4**) red line complexes as χ_M_T vs. T plot. Reproduced with permission from [67]; Published by Wiley, 2015.

Lutzen et al., reported iron(II) spiral architectures of meso-helicate/mesocate rigid Schiff base ligands from 1*H*-imidazole-4-carbaldehyde L_3_ and Pyridine-2-carbaldehyde L_4_ with 1,3-benzene diamine (Figure 5a,b) [67]. The complex designed as [Fe_2_(L_3_)_3_] (**3**) remained high-spin without undergoing SCO, while the complex [Fe_2_(L_4_)_3_] (**4**) remained low-spin up to around 350 K. However, upon heating to above 350 K, the magnetic moment began to increase. At 400 K (the limit of the magnetometer used), a magnetic moment of 1.26 cm^–3^Kmol^–1^ was reached, which indicates that roughly 20% of the metal centers had been switched to the high-spin state. The lack of SCO in complex (**3**) was due to the reason that mechanical strain, imparted by strong CH-π interactions and ligand rigidity (which is locking the bond length of Fe-N) leads to a stabilizing of the high-spin state of the complex (Figure 5c) [68,69].

**Figure 6:**
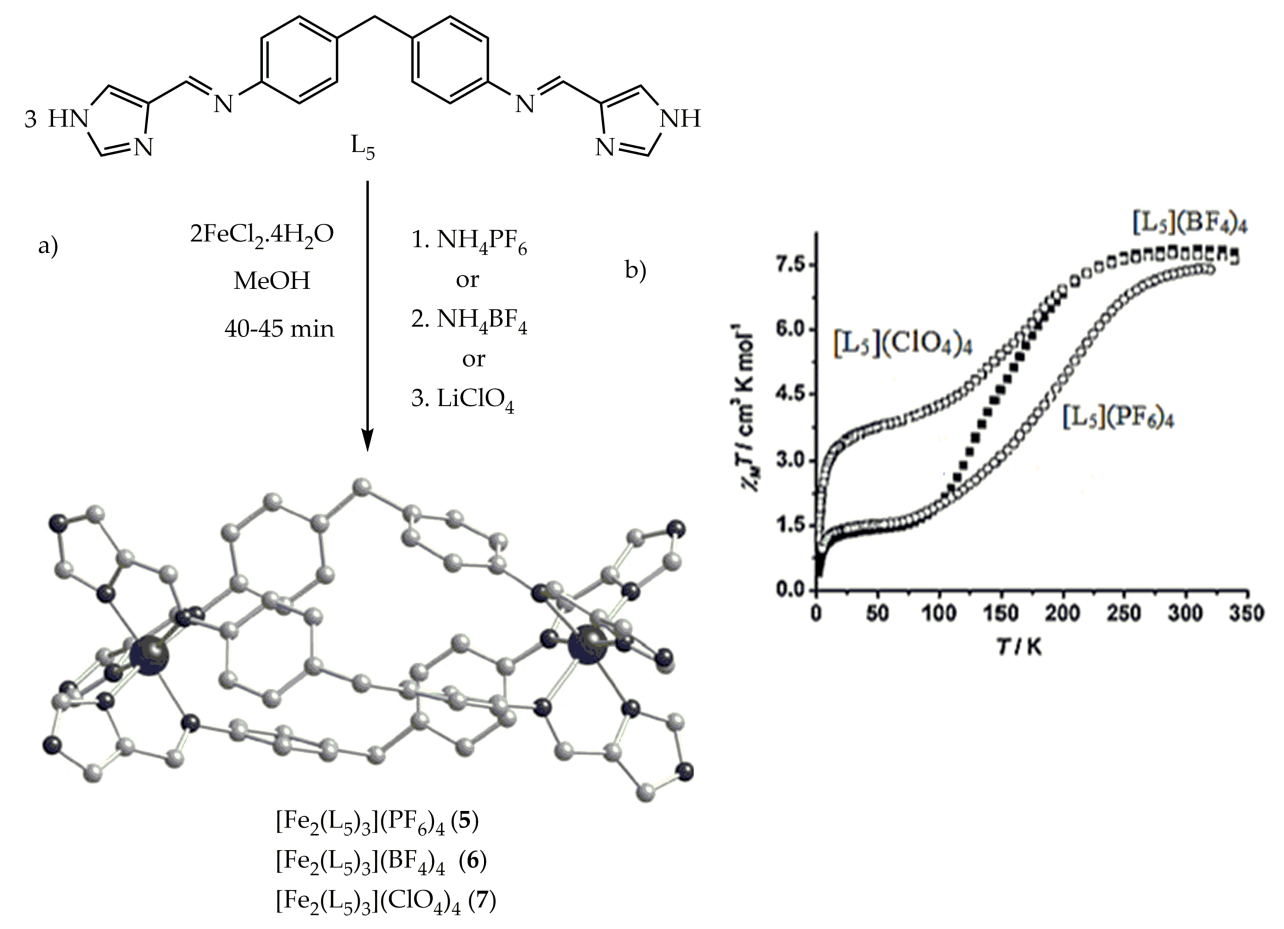
(**a**) The crystal structure of [Fe_2_(L_5_)_3_]X_4_ complexes (X= PF_6_ (5) or BF_4_ (**6**) and ClO_4_ (**7**) (**b**). Magnetic susceptibility χ_M_T vs. T plot for spiral complexes of **5**, **6** and **7** with different counter anions. Reproduced with permission from [70]; Published by Wiley, 2004.

**Chart 2:**
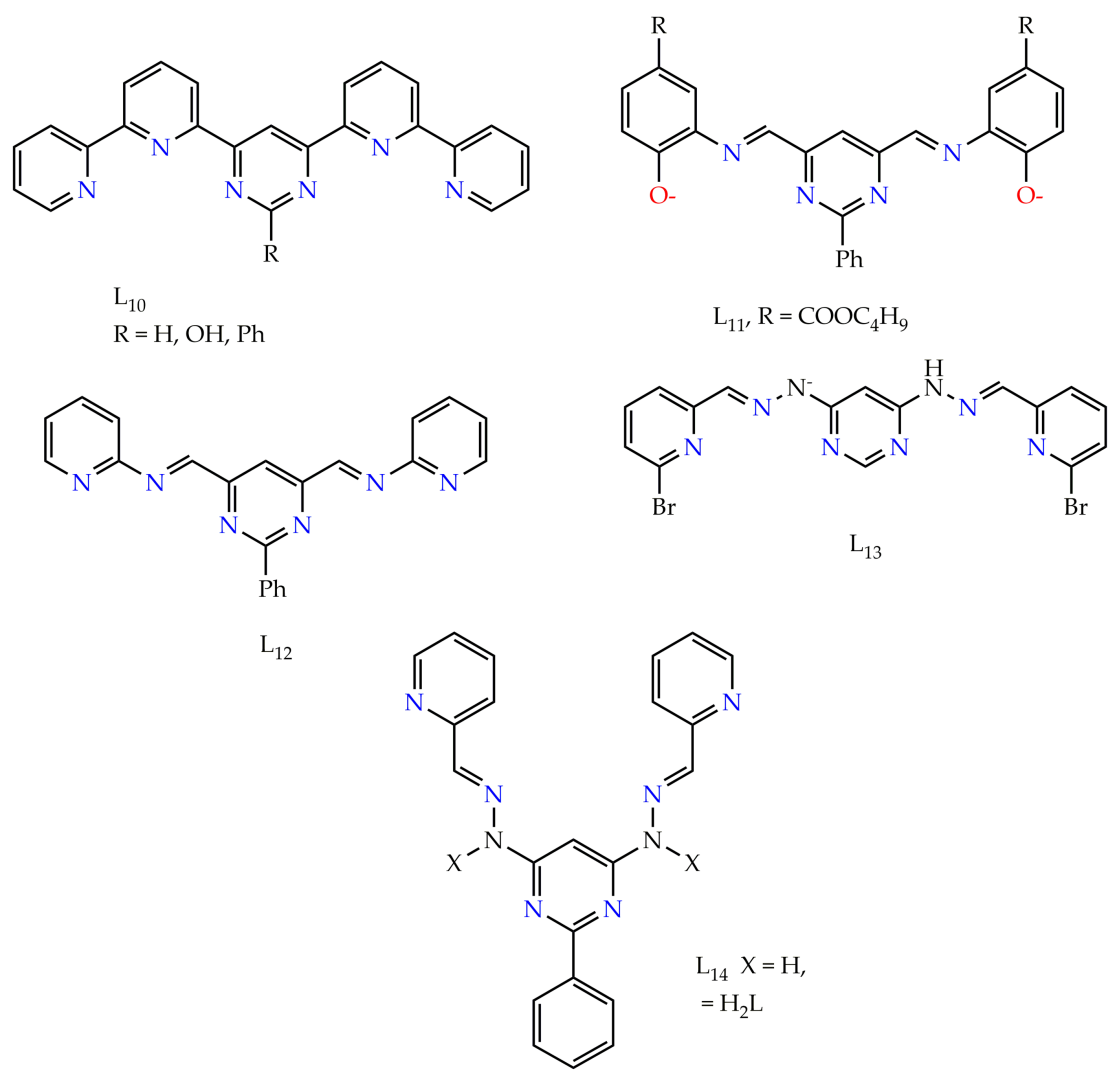
The three bis-terdentate ligands known to form SCO-active Fe_4_L_4_ grids. All ligands provide N_6_ and N_4_O_2_ donor sets to the octahedral iron(II) centers.

The intramolecular and intermolecular interactions such as hydrogen bonding and π-π interactions can affect the SCO which leads to a difficulty in designing cooperative SCO systems. As a result, the same iron(II) spiral architecture can have a different SCO behavior depending on solvent or counter anions. Hannon et al. [70] studied the effects of counter anions such as PF_6_^−^, BF_4_^−^ and ClO_4_^−^ on SCO iron(II) spiral architecture with L_5_ (Chart 2), which has free imidazole N-H groups for hydrogen bonding to solvent and anions (Figure 6a).

The PF_6_^−^ and BF_4_^−^spiral complexes exhibited incomplete SCO at different T_1/2_ values. The χ_M_T value for [Fe_2_(L_5_)_3_](PF_6_)_4_ (**5**) at room temperature was 7.40 cm^3^Kmol^−1^, which corresponds to 2HS iron(II) centers. As the temperature is lowered down to 55 K, the χ_M_T product decreases continuously (Figure 6b), with a slight inflation at around 165 K. At this temperature, χ_M_T is about 3.57 cm^3^Kmol^−1^, which corresponds to what is expected when 50% of the iron(II) ions undergo a thermally induced spin conversion from the HS to the LS. The spiral complex of [Fe_2_(L_5_)_3_](BF_4_)_4_ (**6**) behaves similar to complex (**5**), but the SCO is more rapid. At room temperature, χ_M_T is equal to 7.83 cm^3^Kmol^−1^, which is in the range of values expected for two non-interacting iron(II) centers. The χ_M_T product remains almost constant when cooling to 250 K (Figure 6b) and then decreases rapidly to reach the value of 1.90 cm^3^Kmol^−1^ at 100 K. The plateau observed in the temperature region 70–25 K corresponds to 1.40 cm^3^Kmol^−1^, suggesting an incomplete HS to LS transition with about 20% of the high-spin molar fraction trapped at low temperatures.

For [Fe_2_(L_5_)_3_](ClO_4_)_4_ (**7**), the χ_M_T product is 7.70 cm^3^Kmol^−1^ in the temperature range 340–260 K, consistent with 100% of iron(II) centers in the HS state. As the temperature is lowered, χ_M_T diminishes rapidly to reach a value of 3.81 cm^3^Kmol^−1^ at 60 K (Figure 6). Between 60 and 40 K, the χ_M_T product remains almost constant and finally drops rapidly down to 1.2 cm^3^Kmol^−1^ at 1.8 K. These features reveal an incomplete HS to LS spin conversion occurring in two steps. The first χ_M_T drop is consistent with around 50% of iron(II) ions undergoing HS to LS spin conversion. The second χ_M_T drop may be understood as arising from the combined effect of the electronic SCO and zero-field splitting of the remainder of the HS iron(II) ions. 

**Figure 7:**
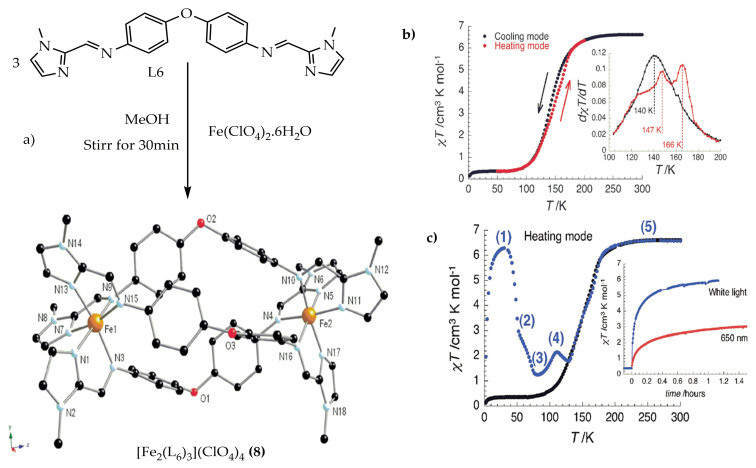
(**a**) Molecular structure of the triple helicate of complex (**8**) (**b**) χ_M_T vs. T plot for **8**.2MeCN at 1000 Oe at sweep rate of 0.6 K/min. Inset: dxT/dT vs. T plot (**c**) Temperature dependence of the χ_M_T product after white light irradiation at 10 K for (**8**). Inset: χ_M_T vs. T plot at 10 K under 650 nm and white light irradiations at 10 K (at T sweep rate of 0.6 K/min). Reproduced with permission from [71]; Published by Royal Society of Chemistry, 2009.

Kruger and co-workers [71] reported the effect of solvents on the SCO behavior of an iron(II) spiral complex of L_6_ (Chart 2). The bis-bidentate L_6_ was synthesized by the methanolic condensation of two equivalents of 1-methyl-2-imidazolecarboxaldehyde and one equivalent of 4,4′-oxydianiline. Then, the corresponding iron(II) bi-nuclear triple helicate complex of [Fe_2_(L_6_)_3_](ClO_4_)_4_ (**8**) was obtained in good yield by stirring three equivalents of L_6_ with two equivalents of Fe(ClO_4_)_2_.6H_2_O in methanol for 30 min (Figure 7a). Complex **8**. 2MeCN showed complete SCO with hysteresis from HS-HS to LS-LS spin states at a low temperature of cooling mode at T_1/2_ = 140 K. However, heating reproduces two-step transitions, which was observed between 147 and 166 K (Figure 7b). Applying white light irradiation between 280 K–10 K, the spectral reflections at solid state were collected. When irradiating with a white light or red light of 650 nm at 10 K, the HS-HS state could be accessed from the LS-LS spin state through LIESST shown in (Figure 7c-inset). The spiral Iron(II) complex showed relaxation behavior in which two different relaxation mechanism were proposed between points of peak 1 and valley 3. By way of heating, the first SCO was observed to a metastable HS-HS spin state at point 4 and, upon heating, could lead to HS-HS at point 5.

**Figure 8:**
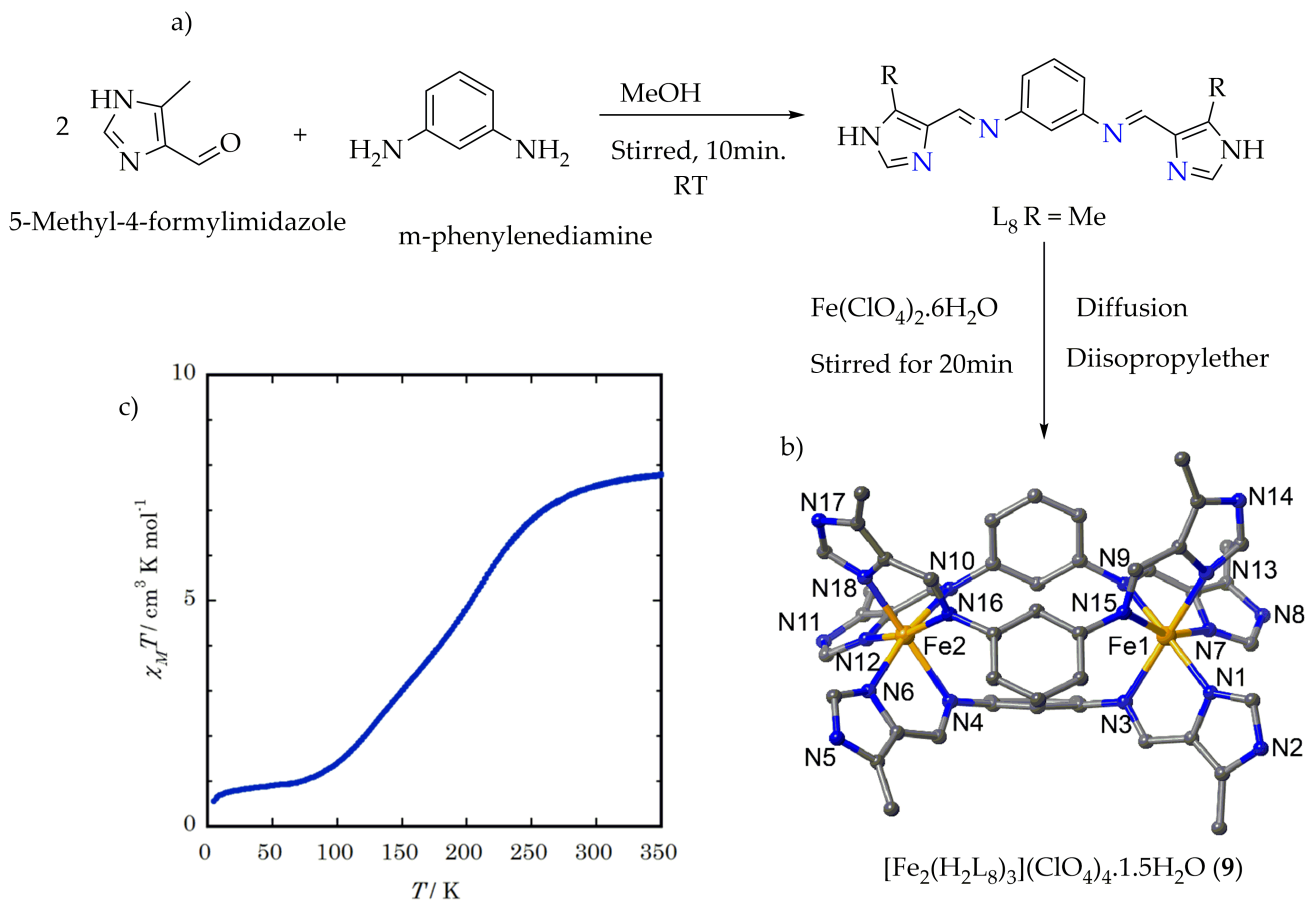
(**a**) Synthesis root of ligand L_8._ (**b**) Crystal structure of the complex **9**.1.5H_2_O. (**c**) The χ_M_T vs. T plot of desolvated complex **9**. Reproduced with permission from **[72]**; Published by Bulletin of the Chemical Society of Japan, 2020.

Sunatsuki et al. [72] studied the effect of substitutions on the SCO behavior of a di-nuclear iron(II) mesocate complex reported by Lutzen et al. [67] by changing the counter anion from BF_4_^–^ to ClO_4_^–^. The bis-bidentate L_8_ (Figure 8a) was synthesized by the methanolic condensation of two equivalents of 5-methyl-4-formylimida-zole and one equivalent of m-phenylenediamine at room temperature. The corresponding triple mesocate complexes of [Fe_2_(H_2_L_8_)_3_](ClO_4_)_4_.1.5H_2_O (**9**) were obtained by stirring three equivalents of L_8_ with two equivalents of Fe(ClO_4_)_2_.6H_2_O (Figure 8b).

The χ_M_T vs. T plot of desolvated complex **9** is shown in (Figure 8c). The χ_M_T value of complex **9** at 350 K is 7.8 cm^3^ Kmol^–1^ which clearly indicates that both iron(II) ions are in the HS states. The χ_M_T values decreased gradually bellow 280 K and reached a plateau near 1.0 cm^3^ K mol^–1^ at 80 K, which is higher than that of the di-nuclear LS iron(II) complex. The χ_M_T value at 80 K shows incomplete (mixed) spin transition with 12.5% 2Fe (HS)-88.5% 2Fe (LS). The χ_M_T measurement of this complex even decreased further below 20 K because of the remaining HS iron(II) ions’ orbital contributions. The SCO in the temperature region of 280 K–80 K shows a two-step spin state transition. The T_1/2_ values for both two steps obtained by the first derivation of the χ_M_T vs. T plot are 212 K and 134 K. There are two possible SCO process for the two-step SCO of this di-nuclear mesocate iron(II) complex, i.e., [LS-LS] ↔ [LS-HS] ↔ [HS-HS] and the process such as [LS-LS] ↔ 50% [LS-LS] + 50% [HS-HS] ↔ 100% [HS-HS]. Generally, it is determined that crystalline complex **9** is in the HS state at 180 K. This fact indicates that the desolvation of crystalline enhances the essential SCO nature of complex **9** cation. The thermal hysteresis is not observed between cooling and heating scan modes of magnetic measurement but show promising SCO behavior comparable with the report of Lutzen et al., (complex **8**), where the similar di-nuclear mesocate shows no SCO behavior and remain HS state [67]. This is due to the fact that the ligand H_2_L_8_ has a suitable ligand-field strength for the SCO of iron(II) due to the introduction of the electron-donating methyl groups on imidazole rings. Generally from the reports, it is clearly shown that by changing the substituent on the ligand, the iron(II) SCO nature can be enhanced specially to get the desired property for better applications.

**Figure 9:**
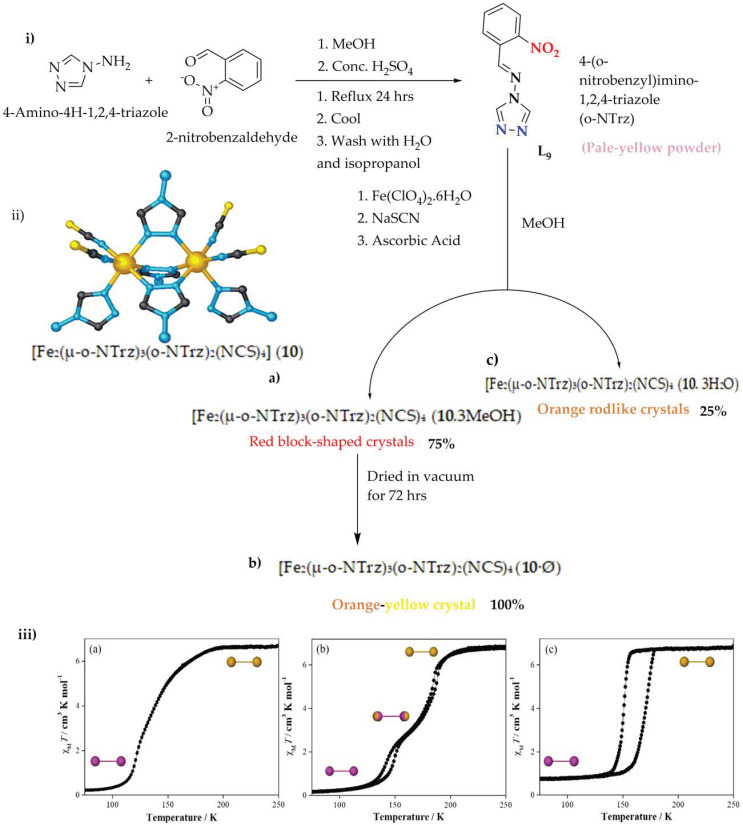
(**i**) *o*-NTrz, 4-(onitrobenzyl)imino-1,2,4-triazole (blue: N-Fe binding groups, red: functionality). (**ii**) Di-nuclear [Fe_2_(μ-*o*-NTrz)_3_(*o*-NTrz)_2_(NCS)_4_] (**10**) core structure (**a**) **10**·3MeOH, (**b**) **10**·Ø, and (**c**) **10**·3H_2_O (**iii**) χ_M_T vs. T plot of the three complexes (**a**) **10**·3MeOH, (**b**) **10**·Ø, and (**c**) **10**·3H_2_O (250−50−250 K; sweep mode; scan rate: 2 K min^−1^). Inset: Spin-state per di-nuclear unit at the indicated plateau region, as determined by structural analysis (Fe-Fe; HS: gold; LS: purple). Reproduced with permission from [82]; Published by American Chemical Society, 2018.

One of the great advantages of di-nuclear iron(II) supramolecular complex is the tendency of adaptability with guests through binding pockets [73,74,75,76,77,78,79] or the intra-spiral cavity [80,81]. Neville et al., studied and reported on the guest adaptability of the SCO properties in iron(II) di-nuclear species underpinned by supramolecular interactions between central metal cation and Schiff base ligand [82]. The corresponding ligand L_9_ (4-(*o*-nitrobenzyl)imino-1,2,4-triazole (*o*-NTrz)) was synthesized from 4-amino-4*H*-1,24-triazole and 2-nitrobenzaldehyde by MeOH condensation (Figure 9i) schematic procedures. Then, the di-nuclear iron(II) Schiff base complex of [Fe_2_(μ-*o*-NTrz)_3_(*o*-NTrz)_2_(NCS)_4_] (**10**) (the solvated (**10** 3MeOH), desolvate (10·Ø) and hydrated (**10**. 3H_2_O) were synthesized from L_9_ Schiff base ligands as observed in Figure 9ii(a–c).

The χ_M_T vs. T magnetic measurements were performed for the three di-nuclear complexes **10**·3MeOH, **10**·Ø and **10**·3H_2_O that show a broad diversity of SCO transition profile characteristics (Figure 9iii(a–c)). The di-nuclear iron(II) Schiff base complex (**10**·3MeOH) in Figure 9 iii(a) shows the χ_M_T value of 6.64 cm^−3^Kmol^−1^ which consistent with two HS iron(II) ions at 250 K. This value remains constant up to 200 K, then shows a gradual decrease in χ_M_T values to 150 K, followed by a more abrupt decrease to 115 K. The χ_M_T values remain constant again at 0.25 cm^−3^ K mol^−1^, below 100 K, which is consistent with two LS iron(II) ions. There is no thermal hysteresis observed over the cooling and heating modes; rather, a gradual and complete one-step SCO transition for **10**·3MeOH is observed at a transition temperature (T_1/2_) of 135 K.

The χ_M_T values of di-nuclear complex (**10**·Ø) (Figure 9iii(a)) remains constant from 250 to 200 K at 6.80 cm^−3^Kmol^−1^, which is inconsistent with two HS iron(II) ions. From 200 to 175 K, the χ_M_T values decrease quickly to 3.39 cm^−3^Kmol^−1^ and then again to 0.25 cm^−3^Kmol^−1^ over the range of 175−125 K, showing a two-step spin transition. The χ_M_T values at intermediate plateau at around 175 K show a 50%HS-50%LS for iron(II) centers. The χ_M_T value 100 K remains constant at 0.25 cm^−3^Kmol^−1^, which indicates the conversion of fully HS to LS iron(II) ions that show complete and two step SCO. Two narrow thermal hysteresis is observed over the heating and cooling mode and the two iron(II) centers for the complex **10**·Ø. One thermal hysteresis is observed at T_1/2↓↑_ 181 and 185 K (ΔT = 4 K), and the second thermal hysteresis is at T_1/2↓↑_ 141 and 148 K (ΔT = 7 K) during 2 Kmin^−1^ scan rate. 

The third di-nuclear iron(II) SCO complex **10**·3H_2_O (Figure 9iii(c)) remains fully HS on the cooling mode from 250 K to 160 K as χ_M_T values remain constant at 6.74 cm^−3^Kmol^−1^. From 160 K to 130 K, the χ_M_T values show a sharp and abrupt decrease to 0.78 cm ^−3^Kmol^−1^, which is responsible for a complete and one-step spin transition to fully LS iron(II) centers. A promising wide thermal hysteresis loop is observed over the cooling and heating mode. The complete and hysteretic one-step SCO transition of **10**·3H_2_O (2 Kmin^−1^) is characterized by T_1/2↓↑_:150, 172 K, ΔT= 22 K. This is the first evidence of the strong cooperativity of metal centers in the complex and one of the widest thermal hysteresis reported so far for a di-nuclear iron(II) SCO compound [74,75,76,77,78,79]. Comparing the three complexes above, all of them show complete spin transition on both cooling and heating modes during the scan rate of 2 Kmin^−1^, but complex **10**·3H_2_O shows a more promising SCO property. Generally, since it is promising research, further studies are recommended by changing external stimuli, guests such as counter anion, and/or substitutions to get a better thermal hysteresis loop as far as the application side is concerned.

### 3.2. Iron(II) SCO Grid-Architectures 

**Figure 10:**
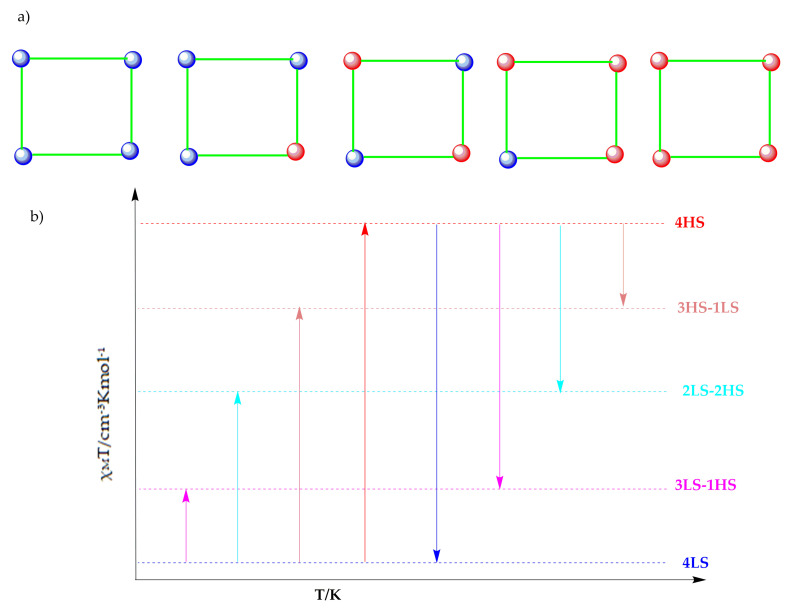
(**a**) Fe_4_L_4_ grid SCO complexes; blue = LS iron(II), red = HS iron(II) and green = N_6_/N_4_O_2_ Schiff base ligands. (**b**) Types of spin state switching in grid complexes.

The quest for novel multinuclear SCO compounds leads to self-assembly, by which grid-like supramolecular architecture SCO compounds could be generated with differences in the spin state of the four metal centers. This tetra-nuclear iron(II) [2 × 2] grid architecture can undergo multiple spin state switching such as [4LS↔(3LS-1HS)], [4LS↔(2LS-2HS)], [4LS↔(1LS-3HS)] and [4LS↔4HS] (Figure 10b) under external stimuli. In addition, effects such as hydrogen bonds, counter ions and solvents have a great role on the modulation of magnetism in these tetra-nuclear iron(II) grid architectures [83,84,85,86,87].

The history of grid-type supramolecular iron(II) SCO complexes starts in early 2000 D.C with Lehn et al., when they tried with 6-(6-(6-(pyridazin-1(6H)-yl)pyridin-2-yl)pyrimidin-4-yl)-2,2′-bipyridine (L_10_) ligand with various substitutions and counter anions, generating grid-like complexes of [Fe_4_(L_10_)_4_](BF_4_)_8_, [Fe_4_(L_10_)_4_](ClO_4_)_8_ and [Fe_4_(L_10_)_4_](PF_6_)_8_ [85,88,89,90]. Unfortunately, from these grid complexes, the SCO properties were not as expected because intramolecular cooperative effects, which were expected from the tetranuclear arrangement of spin carriers, are not evident from temperature-dependent magnetic measurements revealed by very smooth spin conversion. 

**Scheme 2:**
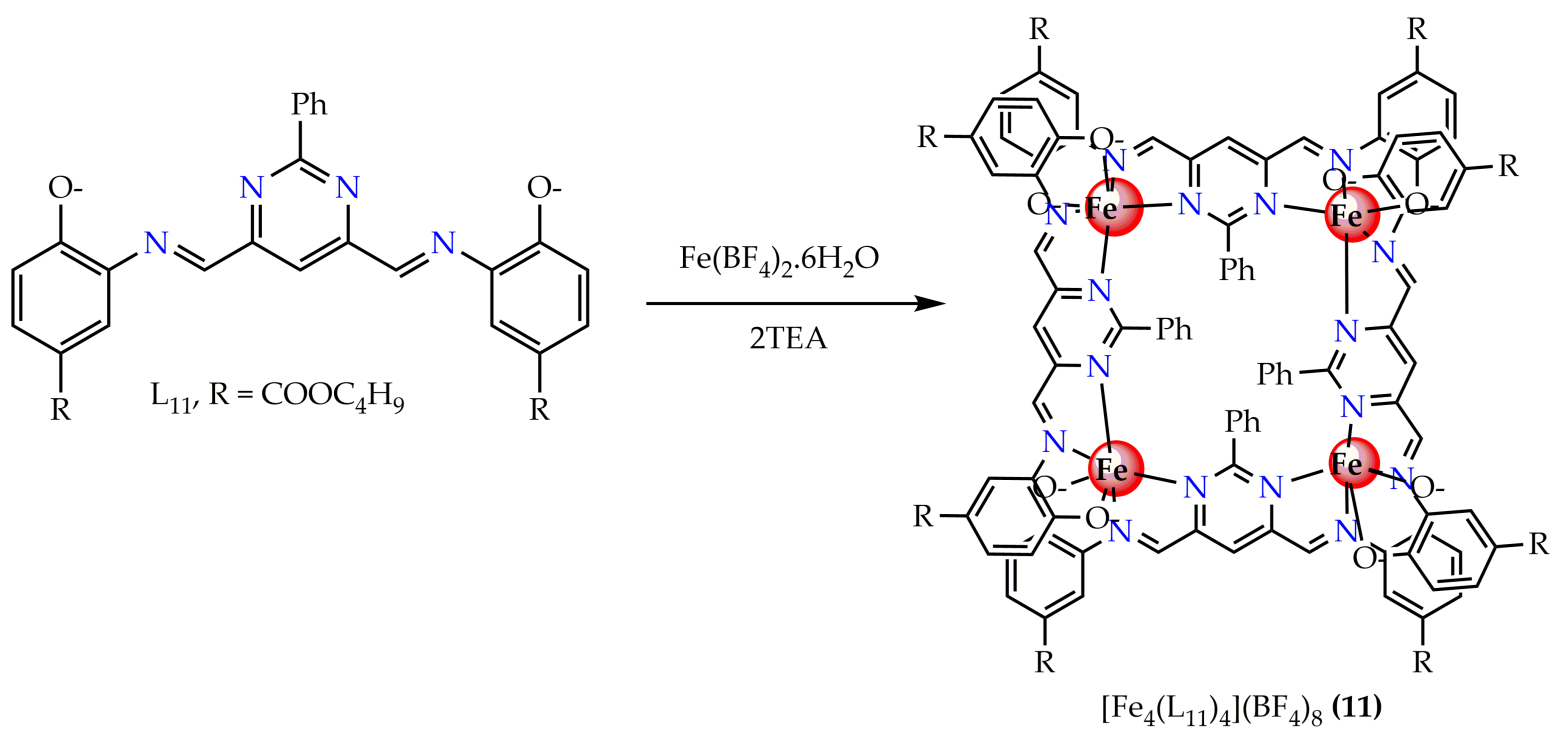
Self-assembly of the [2 × 2] grid-type complex from ligand L_11_ components and iron(II) ions. Reproduced from [87]; published by Wiley, 2009.

In 2009, Stefankiewicz and Lehn [87] reported the first Schiff base complex of Fe_4_^II^ [2 × 2] grid type pseudo octahedral complex [Fe_4_(L_11_)_4_](BF_4_)_8_ (**11**). The neutral grid complex (**11**) was synthesized through the self-assembly of iron(II) cation and L_11_ (diimine ligand) that was formed from the multicomponent Schiff base condensation of phenylpyrimidine dialdehyde with two moles of butoxycarbonyl functionalized 2-aminophenol) (Scheme 2) below [91,92]. The crystal structure of Fe_4_^II^ [2 × 2] grid architecture of this pseudo octahedral iron(II) complex showed an FeN_4_O_2_ coordination environment with four nitrogen atoms from pyrimidine and imine units and two oxygen atoms from phenoxide units. The Fe-N bond lengths were 2.08–2.287 A^◦^ which confirmed the high-spin state of the iron(II) centers since bond lengths can be directly related to the actual spin state of each iron(II) ion [93].

**Figure 11:**
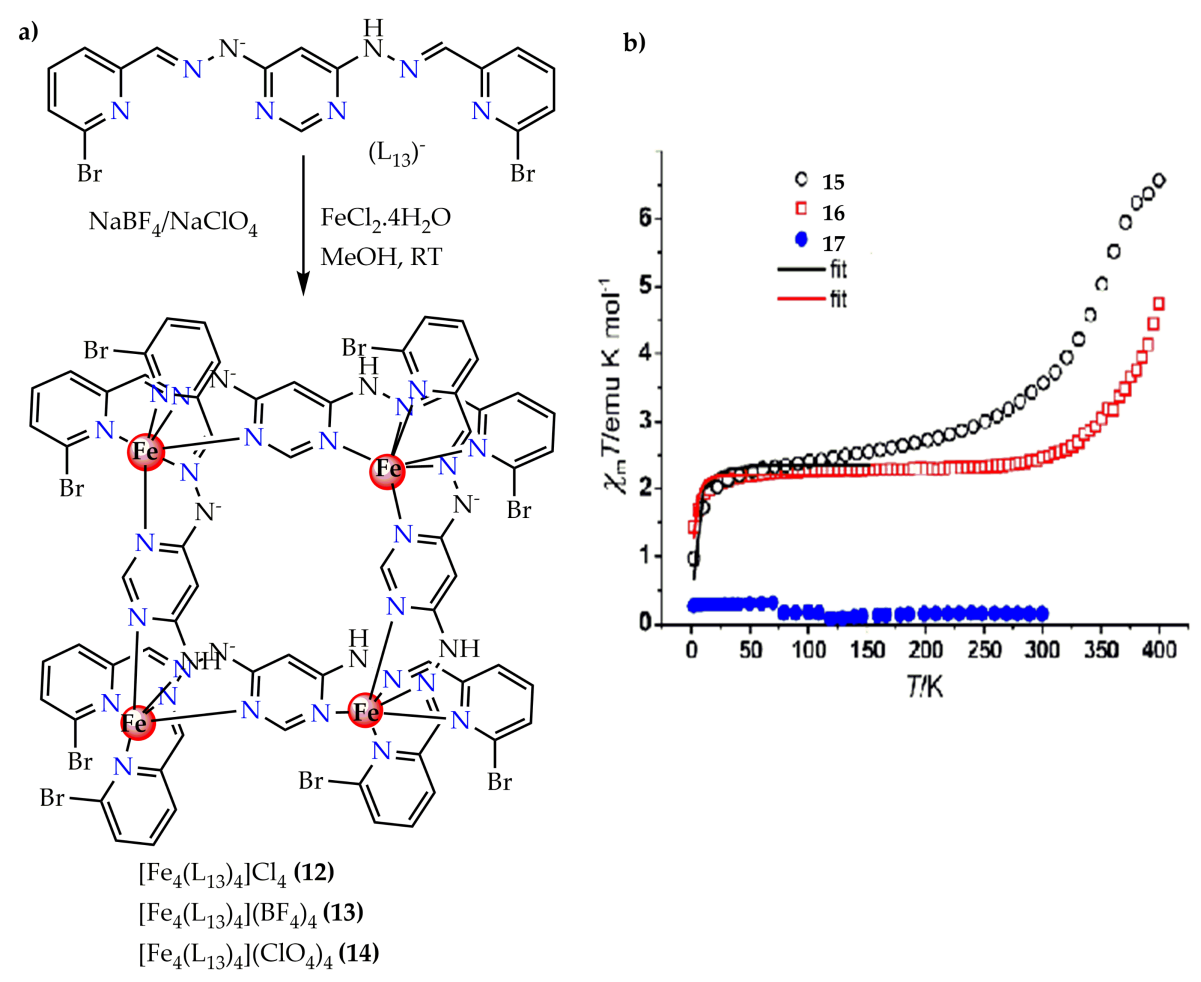
(**a**) Self-assembly synthesis root of complex **15**, **16** and **17** (**b**) χ_M_T vs. T plot for **12** 9H_2_O (black circles), **13** 6H_2_O (red squares) and **14** 4H_2_O (blue) grid complexes. Reproduced with permission from [94], published by American Chemical Society, 2013.

In 2013, Kou et al., reported three SCO grid complexes of [Fe_4_(L_13_)_4_]Cl_4_ (**12**), [Fe_4_(L_13_)_4_](BF_4_)_4_ (**13**) and [Fe_4_(L_13_)_4_](ClO_4_)_4_ (**14**) (Figure 11a) based on a bis(terdentate) Schiff base ligand [(L_13_)^−^, Chart 1] [94]. The SCO for the complex **12** grid is incomplete, with the transition taking place above room temperature between (approximately) [2HS-2LS] and [HS-3LS] states (Figure 11b black data points). The transition is noticeably abrupt, and that of the complex **13** grid is even more abrupt and appears to be centered at about 400 K at the high temperature limit of the measurement (Figure 11b, red data points). Interestingly, the analogous grid with less bulky methyl groups in place of the bromine atoms on the ligand framework is fully LS to high temperature for complex **14** (Figure 11b, blue data points).

**Scheme 3:**
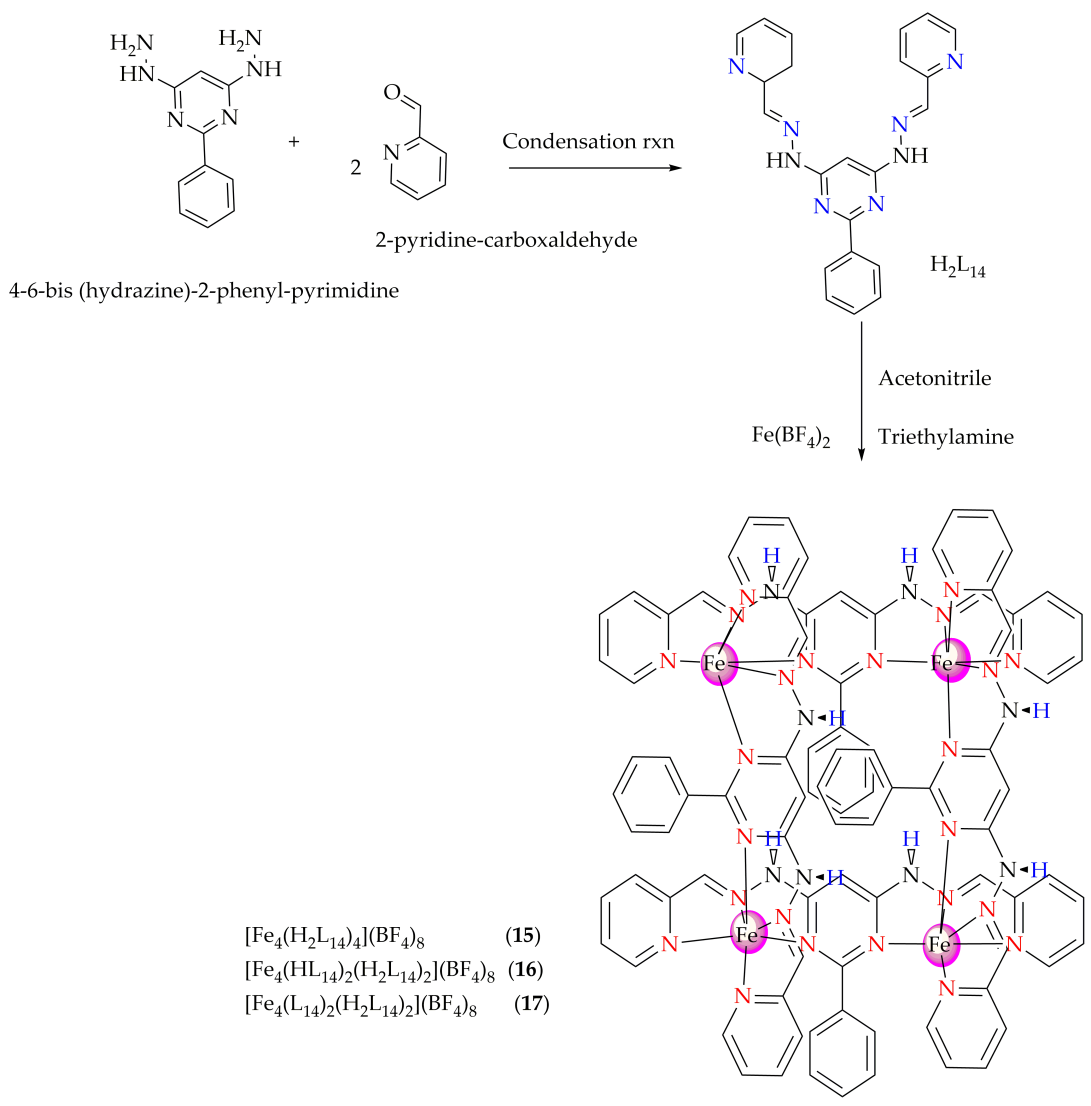
Tetranuclear grid complexes of [Fe_4_(H_2_L_14_)_4_](BF_4_)_8_ (**15**) [Fe_4_(HL_14_)_2_(H_2_L_14_)_2_](BF_4_)_8_ (**16**) [Fe_4_(L_14_)_2_(H_2_L14)_2_](BF_4_)_8_ (**17**). Red spheres represent Fe metal ions. Hydrogen atoms that are colored in blue emphasize the deprotonation sites on the [2 × 2] grid. Reproduced from [95]; published by the American Chemical Society, 2018.

In 2018, Nitschke and Lehn reported on spin state switching in [2 × 2] iron(II) grid complexes (Scheme 3) shown below [95]. The grid was assembled from the hydrazine-based diatopic isomeric ligand (L_14_) (Chart 2). The ligand L_14_ was synthesized through the Schiff base condensation reactions of one equivalent of 4-6-bis (hydrazine)-2-phenyl-pyrimidine with two equivalent of 2-pyridine–carboxaldehyde with two complexation subunits of terpyridine (terpy) ligand. The ligand L_14_ offers the opportunity to synthesize a [2 × 2] iron(II) grid complex and to study the protonic modulation of their physico-chemical properties because of their ionizable N-H states.

**Figure 12:**
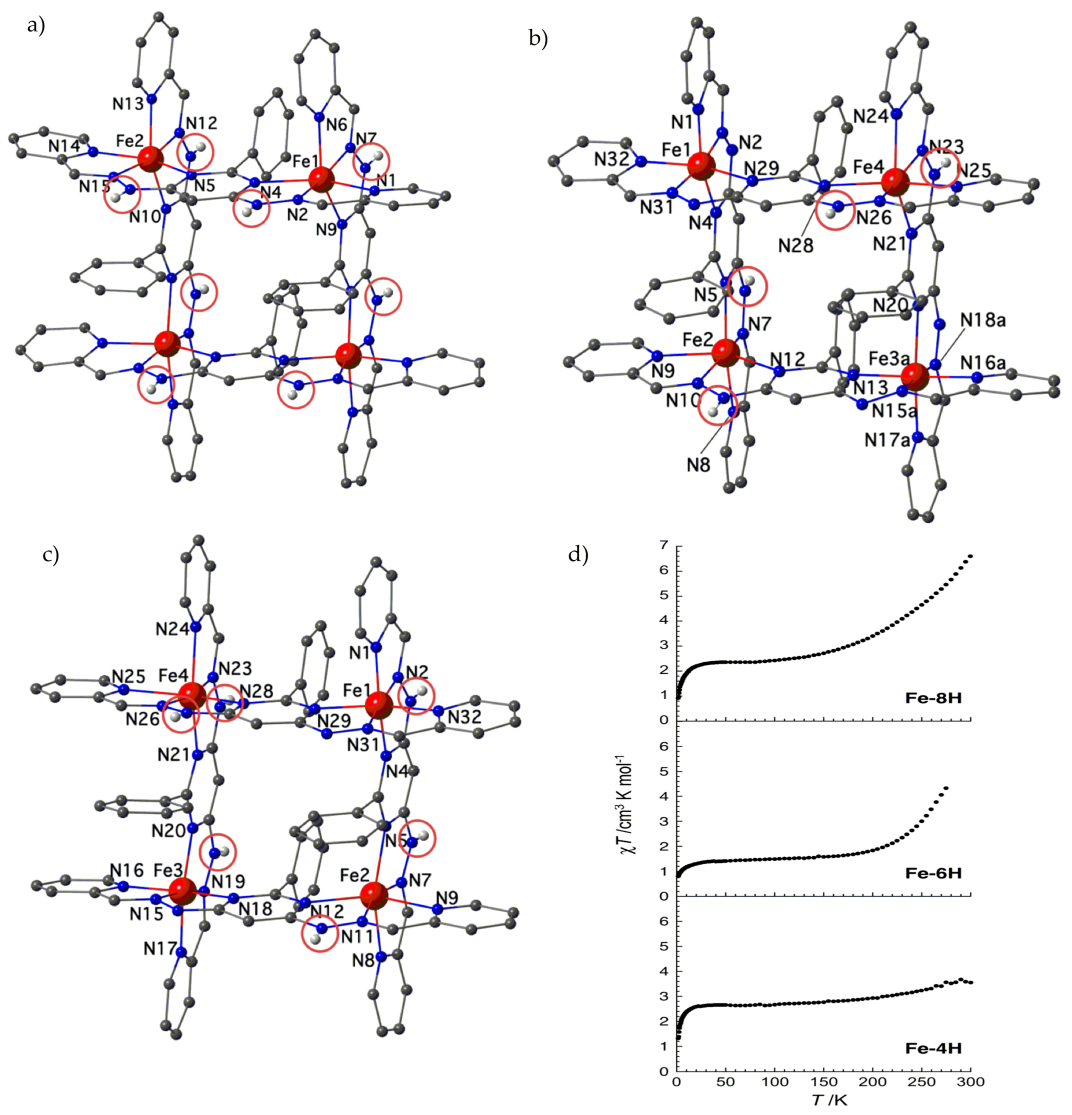
Ball and stick representations of the crystal structures of (**a**) **15**·2MeCN (**b**) **16**·8MeCN (**c**) **17**·12MeCN complexes. (**d**) Temperature dependence of χ_M_T plot of Fe-8H (**15**), Fe-6H (**16**) and Fe-4H (**17**) complexes. Reproduced with permission from [95]; Published by American Chemical Society, 2018.

It is interesting to note that by adding the base trimethylamine to the [2 × 2] iron(II) grid complex of **15** and slightly increasing the temperature by an increment of 2 Kmin^−1^ from 120 K to 290 K helps to observe the different spin states of ionization as shown in Figure 12a–c. The magnetic properties of these complexes show gradual deprotonations of the complexes (Fe-8H (**15**)→Fe-6H (**16**)→Fe-4H (**17**)) with the temperature ranging from 1.85–300 K (Figure 12d). The χT value at the highest temperature for Fe-8H (300 K), Fe-6H (270 K) and Fe-4H (300 K) are 6.6, 4.3 and 3.6 cm^3^ Kmol^−1^, respectively, in which the values are in the region expected for fully HS 4Fe^II^ complexes. This χT gradually decreased during the decreasing of the temperature, reaching a plateau below 120 K for all grids. However, proportions of the HS iron(II) centers Fe-8H, Fe-6H and Fe-4H at room temperature are about 48, 31 and 26% and even at 50 K are 17, 11 and 19%, respectively, which clearly indicates an incomplete SCO for the grid complexes that neither reached a fully 4LS iron(II) at low temperature nor a fully 4HS iron(II) at high temperature (Table 1).

### 3.3. Iron(II) SCO Compounds with Cage Architectures

Supramolecular tetrahedral cages can be self-assembled through coordination-driven self-assembly in which high symmetrical and rigid ligands are typically employed with metal ions that form predictable coordination geometries of improved rational design for the cage. This coordination-driven assembly, sometimes called subcomponent self-assembly, relies on the reversible formation of a metal-ligand bond and a dynamic covalent bond of pryidyl imine or imindazolinmine that is formed by in situ synthesis of aldehydes and amine-factionalized subcomponents [96,97].

**Chart 3:**
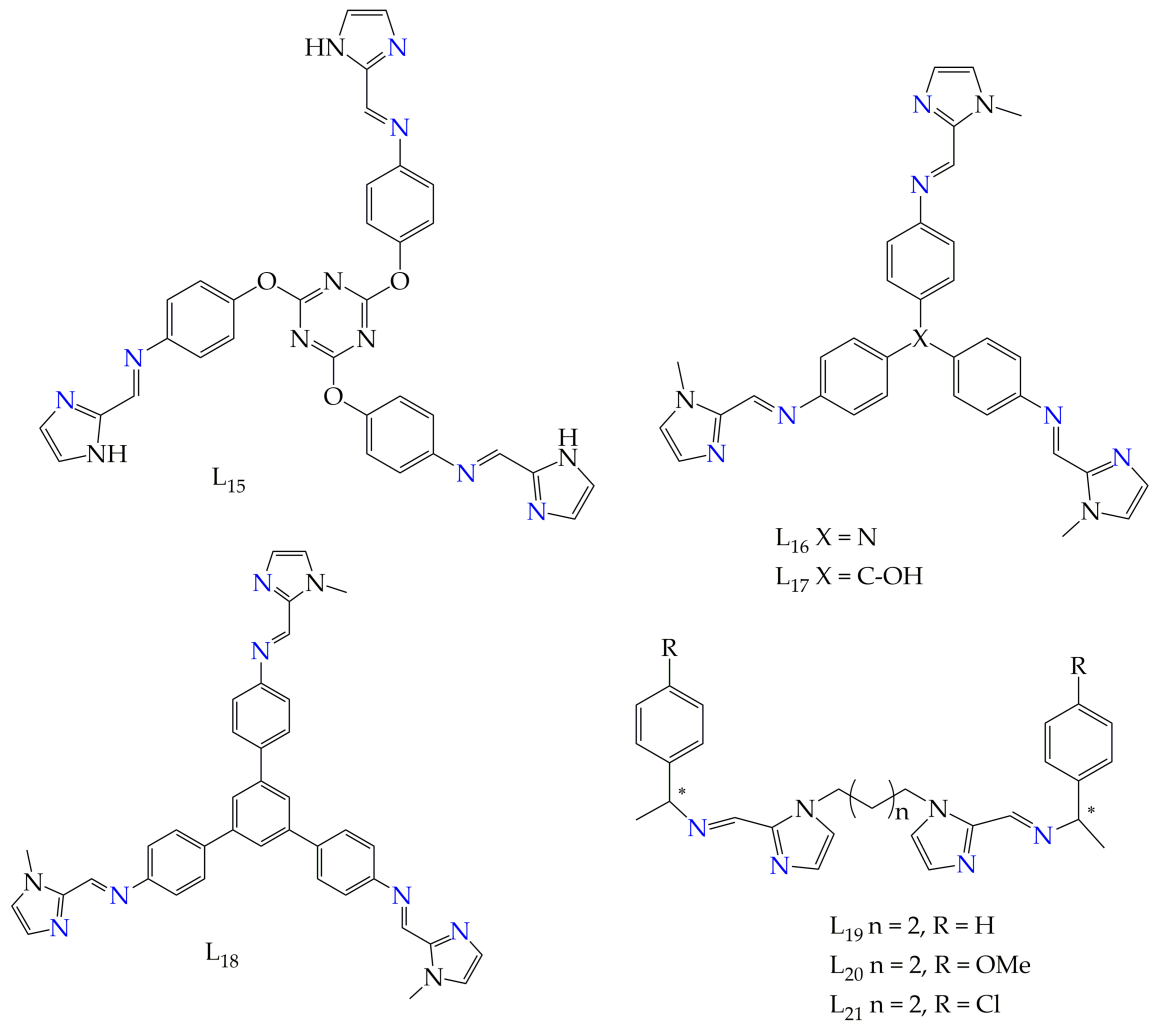
The seven ligands reported in the literature to form SCO-active Fe_4_ tetrahedral cages. Four are tri-topic (face bridging, to give [Fe_4_(L)_4_]^8+^) and the other three are di-topic (edge bridging to give [Fe_4_(L)_6_]^8+^). All provide N^6^ donor sets to the octahedral iron(II) metal ions through the binding of three sets of bidentate binding pockets. For L_19_–L_21_ both the (R, R) and (S, S) configurations at the chiral (*) center have been used to form cages.

Tri-topic and di-topic bidentate ligands, as shown in Chart 3, have been used in iron(II) mediated supramolecular tetrahedral cages, which are mostly synthesized through self-assembly; these architectures were SCO active in the systems. Designating iron(II) cage architectures for the purposes of SCO is not an easy task since it is very crucial in controlling the ligand field strength around Fe_4_ of iron(II) metal centers of the cage. Due to this reason, all iron(II) cage architectures reported so far for the purposes SCO properties depend on imindazolimine-based ligands, as they have a weaker field strength than pyrimidine and 2,2′-bipyridyl based ligands that are typically used in low-spin iron(II) supramolecular cages. The advantage of iron(II) supramolecular cages is that they show better SCO behavior than the spiral architecture due to the better possibility of guest recognition by the cage interior modifications [49,80].

**Figure 13:**
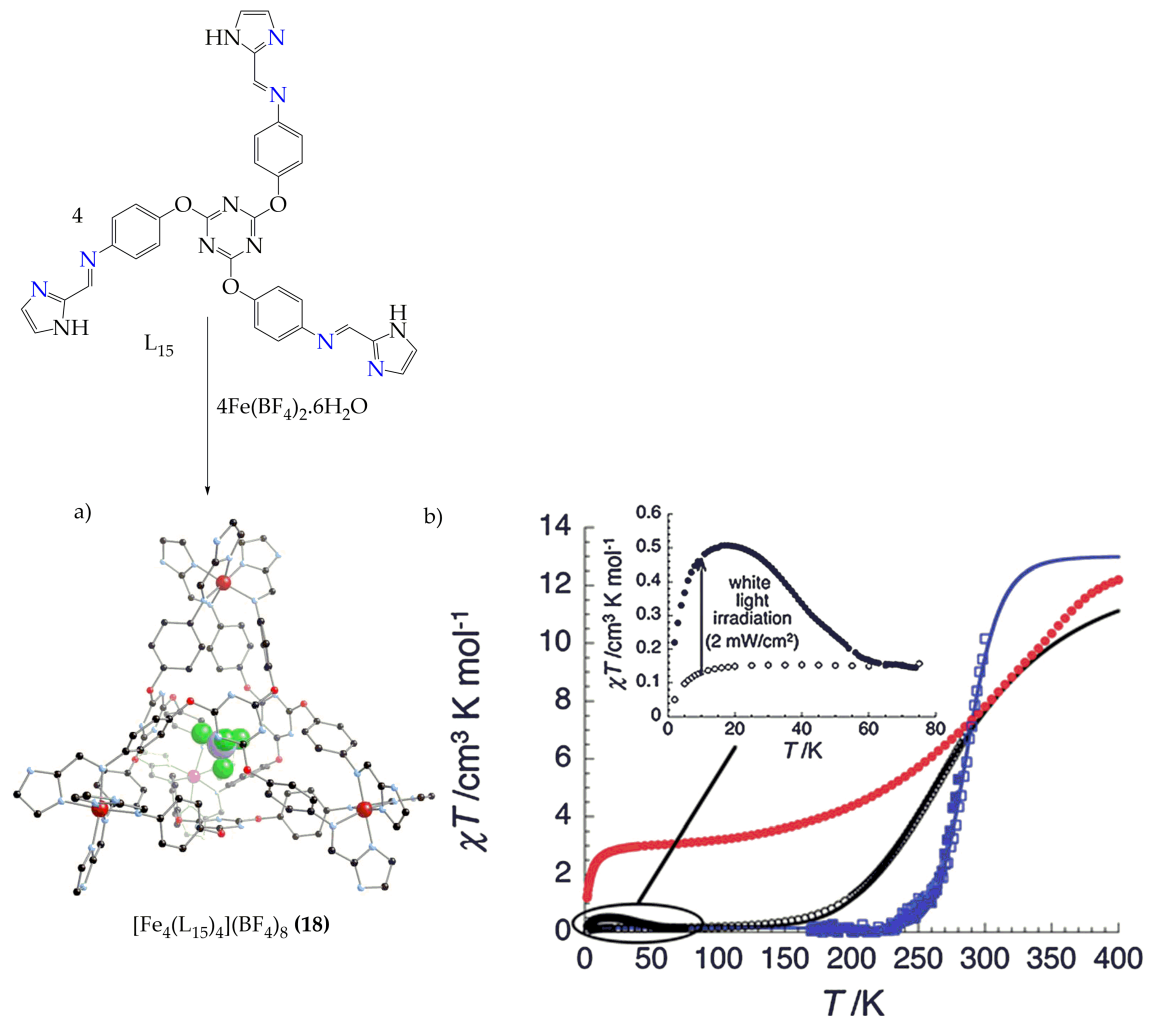
(**a**) Structure of complex **18** showing the encapsulated BF_4_ counter anion in space fill; (**b**) *χT* vs. *T* data of Complex **18.** Reproduced from [96]; Published by Royal Society of Chemistry, 2013.

In 2013, Kruger et al., reported the first example of tetrahedral cage iron(II) SCO active architecture [96]. The self-assembly of this architecture was through a face capping ligand of the ligand L_15_ from triamine with a substituted triazine moiety and 2-imidazole-carboxaldehyde. This iron(II) architecture resulted in [Fe_4_(L_15_)_4_](BF_4_)_8_ (**18**) tetrahedral architecture (Figure 13a) which showed SCO in both a solid and solution state. For complex **18**, at 290 K, the χT product is 7.4 cm^3^Kmol^−1^, which is lower than the expected value for four HS iron(II) ions, suggesting that the cage is ~60% HS at this temperature. The χT product gradually decreases as the sample is cooled and is complete at ~120 K, leaving a complete LS state for the cage with a small residual paramagnetic signal (at 60 K: 0.15 cm^3^Kmol^−1^) observed (black open dots, Figure 13b). 

When complex **18** is dissolved in acetone, the SCO properties probed by magnetic susceptibility are clearly retained. As shown in Figure 13b (blue squares), the χT product of this solution is 10.1 cm^3^Kmol^−1^ at 300 K and exhibits a complete low-spin state below 220 K. The magnetic susceptibility of a dried sample of complex **18** was also measured, allowing measurements up to 400 K (red trace, Figure 13b). The χT product at 400 K is 12.4 cm^3^Kmol^−1^; however, the shape of the χT curve suggests the cage is not quite in its complete HS state even at this higher temperature. As the temperature is lowered, the χT product again gradually decreases before reaching a plateau at ~60 K. The χT product at 60 K is 3.08 cm^3^Kmol^−1^, suggesting that one iron(II) center remains in the HS state.

**Figure 14:**
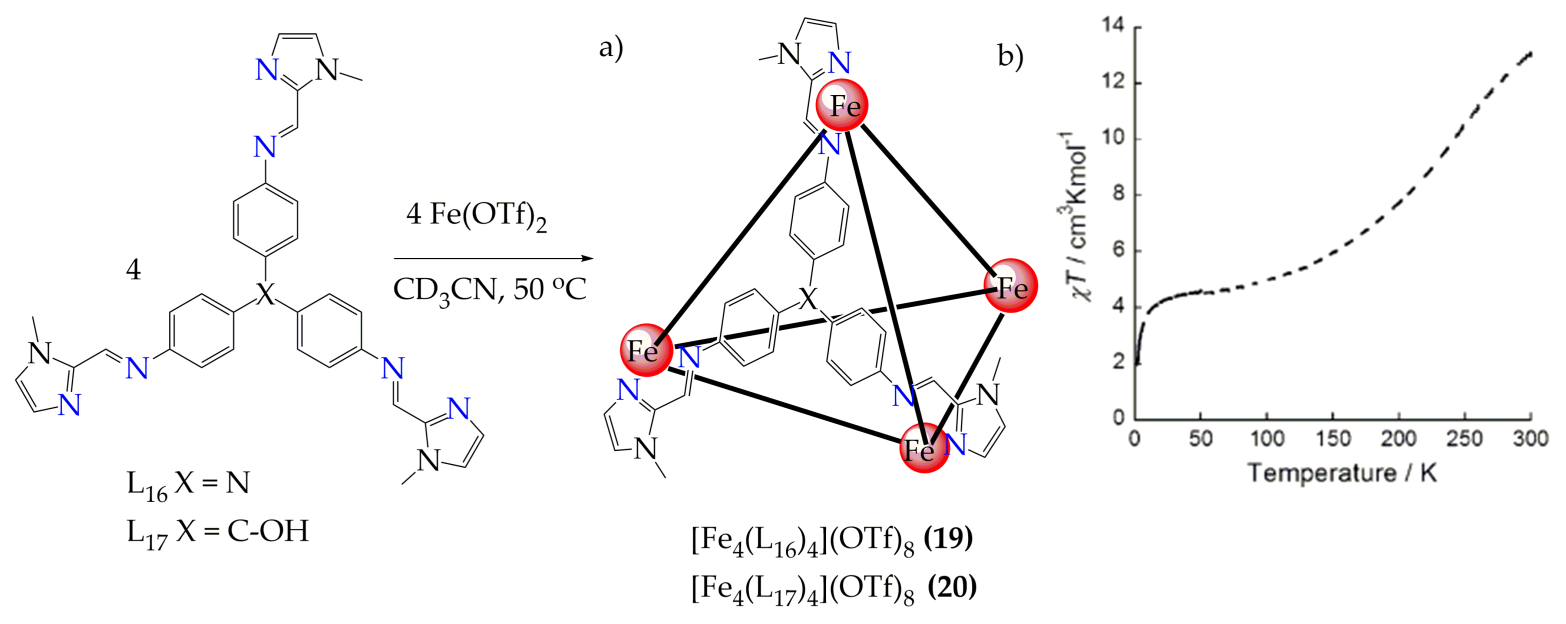
(**a**) Representation of the molecular structure of tetrahedral cage complexes of **19** and **20.** (**b**) Temperature dependence of χT of **26**, between 300–2 K. Reproduced with permission from [98]; Published by Wiley, 2013.

In 2013, Nitschke et al., synthesized smaller face-capping on a pair of iron(II) mediated Fe cages [Fe_4_(L_16_)_4_](OTf)_8_ (**19**) and [Fe_4_(L_17_)_4_](OTf)_8_ (**20**) through the self-assembly of iron(II) with L_16_ and L_17_ (Figure 14a) and investigated guest binding influence on SCO [98]. Both supramolecular complexes were SCO-active in nitromethane, but complex **19** was SCO-active in the solid state only having [4HS] to [HS-3LS] spin transitions. In addition, the SCO properties of these complexes were studied by modification through encapsulations of anionic Br- and neutral CS_2_ as a guest. Encapsulations lowered the T_1/2_ from 336 K for the empty cage of complex **20** to 328 K for Br^−^ guest binding and 324 K for CS_2_. The encapsulations of larger guests (CS_2_) changed the geometry of the host architecture to a greater extent than the smaller guest (Br^−^) and increased the HS state at the same temperature. 

**Figure 15:**
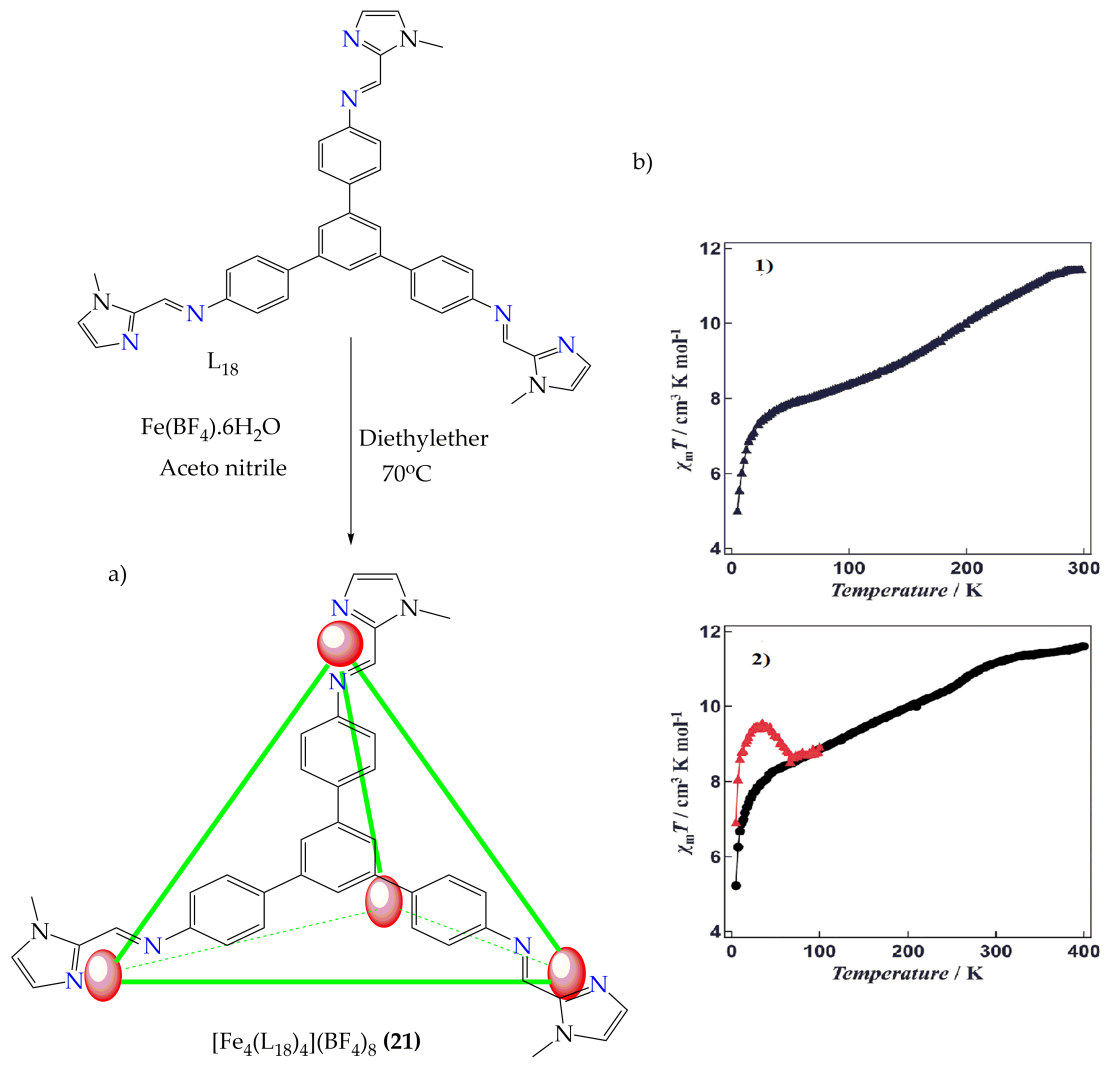
(**a**) Complex cation structure of **21** (red spheres = Fe^II^ centers, green = L_19_); (**b**) χ_m_T vs. T plots for b (1) solvated **21**.16MeCN, the sample was measured in the temperature range between 5 K and 300 K and b (2) non-solvated **21** compound. Reproduced from [99]; Published by Royal Society of Chemistry, 2015.

In 2015, a tetrahedral cage from L_18_, [Fe_4_(L_18_)_4_](BF_4_)_8_ (**21**) (Figure 15) was reported by Li et al. [99], which was a face-capped SCO-active aggregate. It was synthesized through self-assembled Schiff base from an imidazole carboxaldehyde and a trigonal planar triamine (Chart 3). The amines were connected by X (X = N or C-OH) which kept the angle of 120^o^ making the ligand more rigid to form a tetrahedral supramolecular architecture through face-caped connectivity to the four iron (II) centers. The magnetic susceptibility measurements reveal a gradual incomplete spin transition between 5 and 300 K. The SCO phenomenon between the HS and LS states for solvated **21** 16CH_3_CN and non-solvated **21** was conducted by measuring the molar magnetic susceptibility χ_M_ as a function of temperature T (Figure 15b (1 and 2)), respectively. The χ_M_T value for **21** 16CH_3_CN is equal to 11.44 cm^3^Kmol^−1^ at 300 K and 7.85 cm^3^Kmol^−1^ at 50 K, respectively, consistent with a gradual spin-crossover transition. After annealing at 400 K, non-solvated compound **21** was obtained. The χ_M_T value of the desolvated material is equal to 11.60 cm^3^ Kmol^−1^ at 400 K, suggesting iron(II) is in the high-spin state. On cooling, the χ_M_T values gradually decrease (Figure 15b (2)). The χ_M_T value at 50 K is equal to 8.29 cm^3^Kmol^−1^, which shows that spin-crossover from the high-spin to the low-spin states is induced in about 30% of the iron(II) ions.

**Figure 16:**
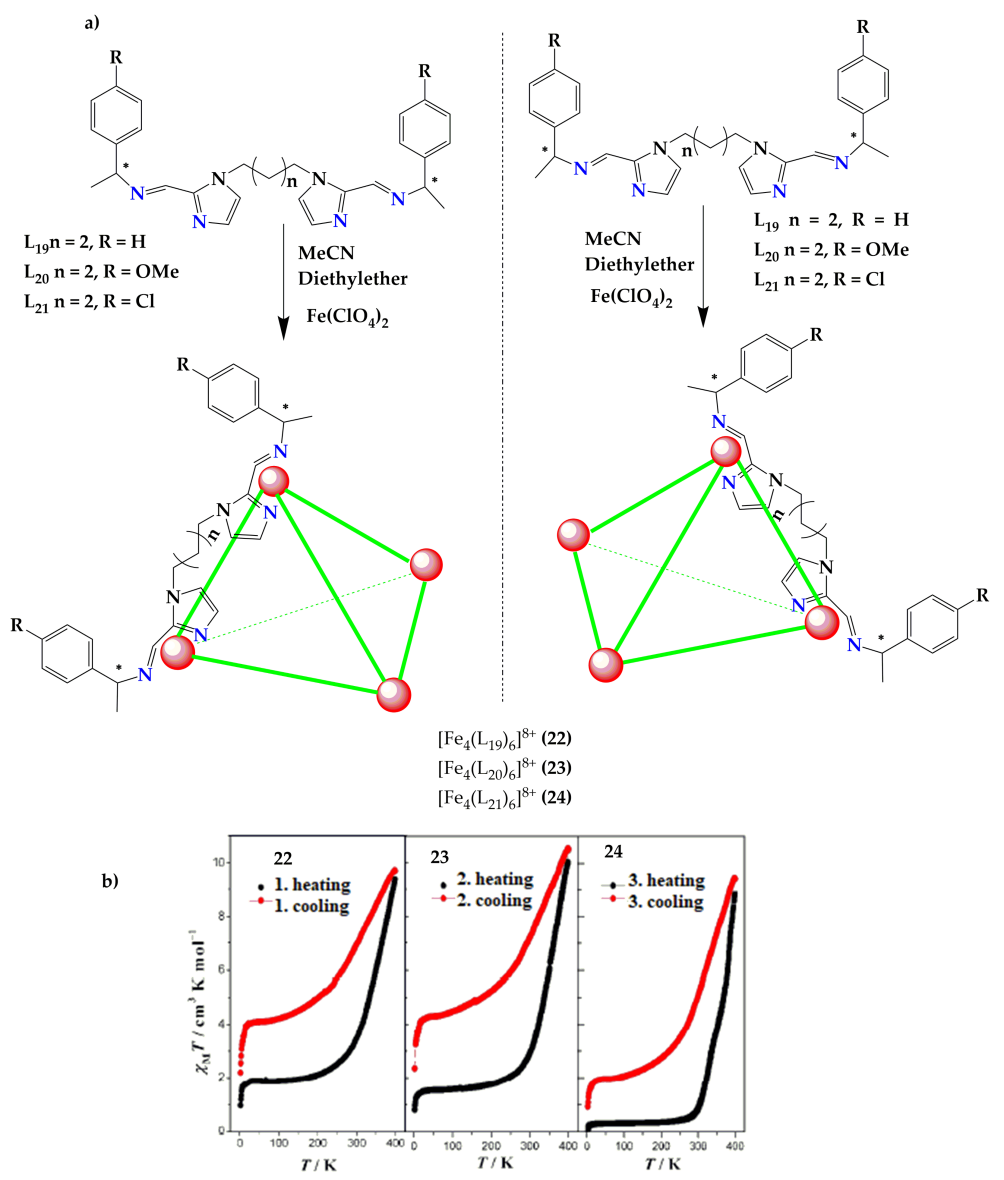
(**a**) Enantiopure tetrahedral cages of complex **22**, **23** and **24** synthesized through subcomponent self-assembly of 1,4-di(imidazole-2 carboxaldehyde) butane chiral phenylethylamine and iron(II) ions. (**b**) Plots of χ_M_T vs. T for cage complexes of **22**, **23** and **24.** Reproduced with permission from [48]; Published by Royal Society of Chemistry, 2015.

Gu et al., reported on tetrahedral iron(II) cages based on di-topic Schiff base bidentate ligands L_19_-L_21_ (Chart 3) which defines the six edges of the tetrahedron, giving supramolecular complexes of [Fe_4_(L_19_)_6_]^8+^ (**21**) [Fe_4_(L_20_)_6_]^8+^ (**23**) and [Fe_4_(L_21_)_6_]^8+^ (**24**) (Figure 16a) [48]. This iron(II) enantiopure homochiral tetrahedral cage was synthesized through a self-assembly system that involved either the (R) or (S) chiral complex of the tetrahedral cage that showed an optical isomer of the amine. These enantiomer complexes exhibit identical magnetic properties. The variations in R groups that attached to the ligand (R = H, OMe or Cl) and solvent desorption are factors that slightly affected the SCO behavior (gradual and incomplete) in the solid state. The cages are 3-18% HS when solvated and 17–37% HS when desolvated at 200 K and spin state transition to 74-87% HS at 400 K desolvated and best stabilized when R = Cl with the LS state in the architecture.

### 3.4. Iron(II) SCO Compounds with Cubic Architectures

**Chart 4:**
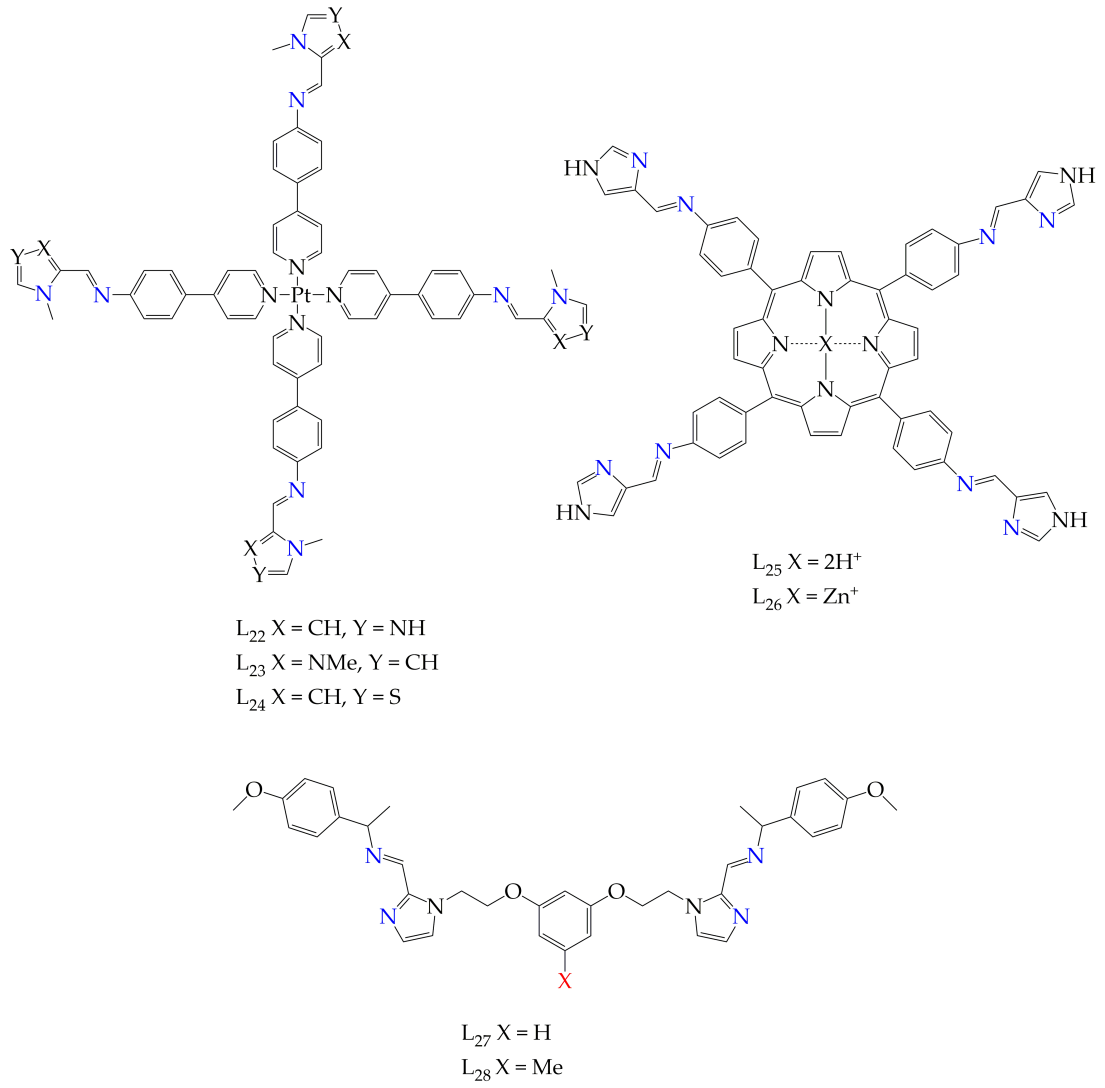
Ligands to form cubic supramolecular architectures of iron(II) SCO complexes.

As structural diversity and complexity is increasing in metallo-supramolecular compounds, it is difficult to achieve various geometries by a monometallic-ligand based building block approach [100,101]. So, new strategies have been developed in constructing hetero bimetallic complexes. This includes the implementation of new designs by way of complex-as-a-ligand (ligand that has metal connectivity at its center to keep it rigidity) strategy, leading towards hetero-bimetallic cubic architectures [102]. Moreover, in the search for new functional materials, hetero-bimetallic aggregates offer the exciting chance to combine properties of two different metal centers within one discrete structure, potentially expanding their electrochemical, photo-physical or magnetic properties [103]. Until now, only very few examples of spin crossover in cubic architectures are known in the literature. This might be due to the fact that classic covalent ligand synthesis to achieve the formation of such structures are often challenging, which in turn limits the possibility to investigate the influence of different ligands on spin-crossover centers [104,105]. Chart 4 shows the examples of Schiff base ligands that might leads to the formation of cubic architecture. 

**Figure 17:**
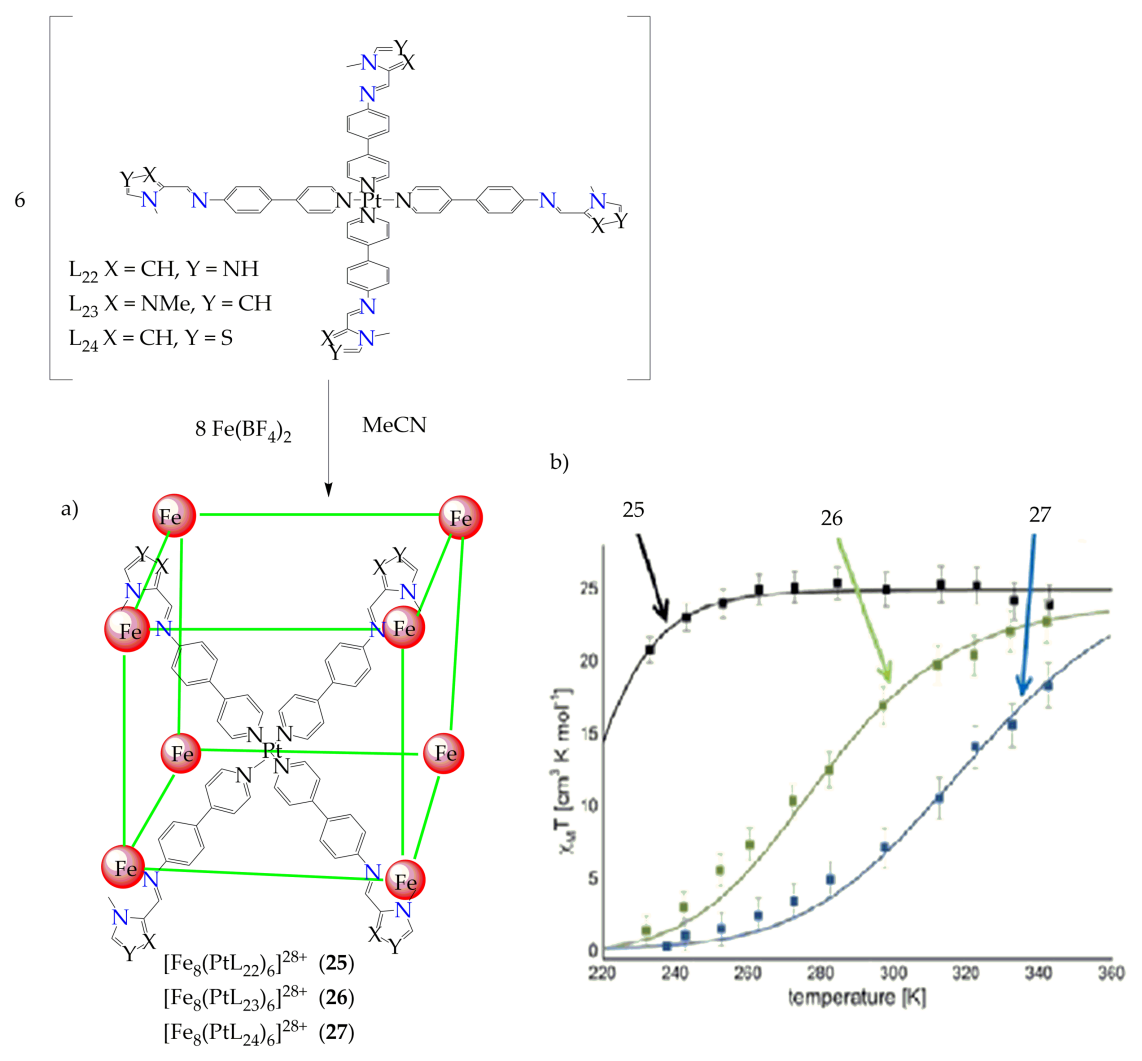
(**a**) Hetero-bimetallic cubic architecture of (**a**) [Fe^II^_8_(PtL_22–24_)_6_](BF_4_)_28_ and (**b**) plots of χ_M_T vs. T for cubic cages of [Fe^II^_8_(PtL_22–24_)_6_](BF_4_)_28_. Reproduced with permission from [104]; Published by Wiley 2021.

In 2012, Nitschke et al., reported on hetero-bimetallic cubic cages with the general formula [Fe_8_(PtL_22–24_)_6_]^28+^ of L_22_–L_24_ (Figure 17a) [106]. This cubic architecture has eight iron(II) centers that coordinated to pyridylimine binding sites with iron centers in the diamagnetic low-spin state. The iron(II) cations were bridged with Pt(II) based ligands by the face-capping tetravalent building blocks through self-assembly. The synthesis of this cubic architecture was made by combinations of precursors such as Pt(II) building blocks with 2-formyl pyridine as an azine component with iron(II) salt. 

Lutzen et al., 2021 extended the concept previously established by Nitschke and co-workers in which the switchable SCO iron(II) supramolecular cubic architecture was resynthesized to strength the idea of spin state switchability through the exchange of the azine building blocks of the subcomponent self-assembly step with suitable azole types of ligand facilitating to reduce ligand field strength [104]. The reduced ligand field stabilizes the paramagnetic HS state in iron(II) cations and facilitates SCO behavior [107,108]. The three ligands (L_22_–L_24_) are directly related to each other and can easily be obtained from the same starting material, employing a simple subcomponent self-assembly strategy. In the new three iron(II) mediated supramolecular cubic architectures of [Fe_8_(PtL_22_)_6_]^28+^ (**25**), [Fe_8_(PtL_23_)_6_]^28+^ (**26**) and [Fe_8_(PtL_24_)_6_]^28+^ (**27**) in Figure 17a with SCO behavior, two of them showed spin transition centered nearly at room temperature and one at low temperature [109].

The supramolecular complex **25** synthesized from a 4-imidazolyimine analogue of L_22_ (Chart 3) shows as purely paramagnetic at room temperature [90]. The HS state was found to be stable down to a temperature of 245 K, but by lowering the temperature even further, the SCO process of this complex observed at 233 K which translated to roughly 85% iron(II) centers still in the paramagnetic HS state. Due to the limitation set by the freezing point of acetonitrile, checking the SCO process to lower temperatures was difficult, but the spin transitions of the L_22_-based architecture was predicted at T_1/2_ = 215 K by the ideal solution model (SI).

The iron(II) Schiff base complex **26** that assembled from N-methyl-2-imidazolyimine analogue L_23_ at room temperature indicated the presence of both the HS and LS state, in which the HS state value corresponded to 63% of the iron(II) centers. The spin transition temperature of this cubic architecture was slightly below room temperature, with a T_1/2_ = 281 K. Due to the temperature limitations set by the solvent, the situation could not be observed purely whether it was a paramagnetic or diamagnetic complex. Increasing the temperature to 343 K showed 84% HS, but lowering the temperature to 233 K showed very close to fully diamagnetic properties. The 4-thiazolyimine analogue ligand L_24_, which forms complex **27**, showed 77% of LS iron(II) centers at room temperature. At high temperatures, the fraction of HS centers becomes significantly larger and the complex showed an SCO of the highest spin transition temperature of T_1/2_ = 324 K. 

**Figure 18:**
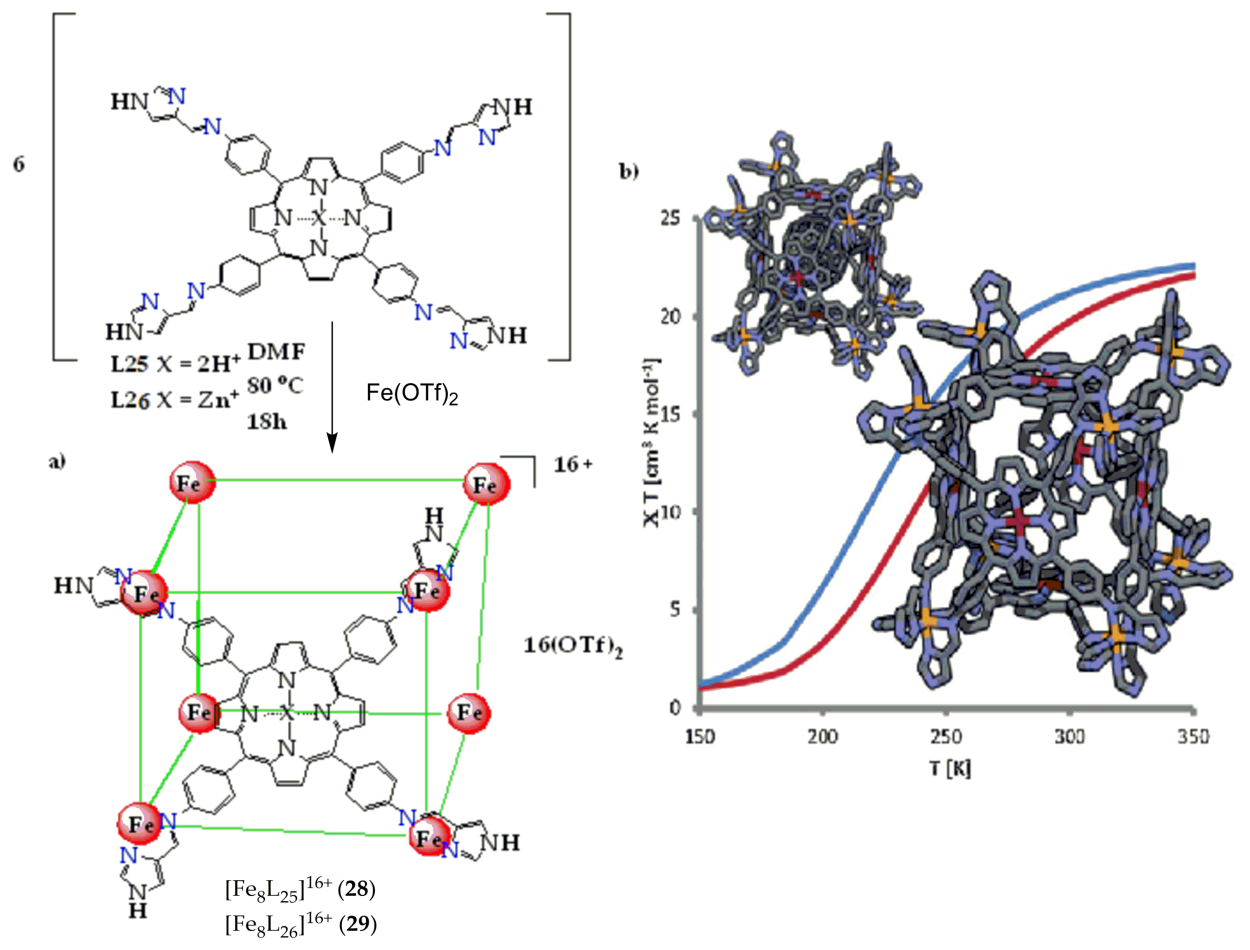
(**a**) Hetero-bimetallic cubic architecture of [Fe_8_L_25_]^16+^ (**28**) and [Fe_8_L_26_]^16+^ (**29**) (**b**) χ_M_T vs. T plots of C_70_ guest encapsulated (**top**) and empty cavity (**bottom**) complex **29.** Reproduced with permission from [105]; Published by Wiley, 2017.

In 2017, Lutzen et al., reported two iron(II) cubic architectures of the general formula [Fe_8_L_(25–26)_]^16+^,which exhibited SCO in a methanol medium [105]. The supramolecular cubes were constructed from eight iron(II) centers and six-tetra-topic porphyrin ligands L_25_ and L_26_ (Chart 3) to form [Fe_8_L_25_]^16+^ (**28**) and [Fe_8_L_26_]^16+^ (**29**) in which the porphyrins were all either uncoordinated (L_25_) or bound to Zn(II) of (ZnL_26_)—acting as a face-capping unit (Figure 18a). Zn(II) was coordinated to the porphyrin ligand faces of eight iron(II) in the cubic form and stabilized the HS state very slightly. The Zn-coordinated species was studied by Evans method from 298 K–203 K in deuterated-methanol, revealing that the transitions occurred from 85% HS to 30% HS, while the cube without Zn(II) transitions occurred from 80% HS to 15% HS. However, the guest encapsulation of fullerene (C_70_), which was facilitated by the appropriate internal cavity of the host, had a great influence on SCO, was found to stabilize the HS state and lowered the T_1/2_ of the solution of SCO by 15 K and 20 K for L26 and L_25_ based 8Fe cubes respectively.

**Figure 19:**
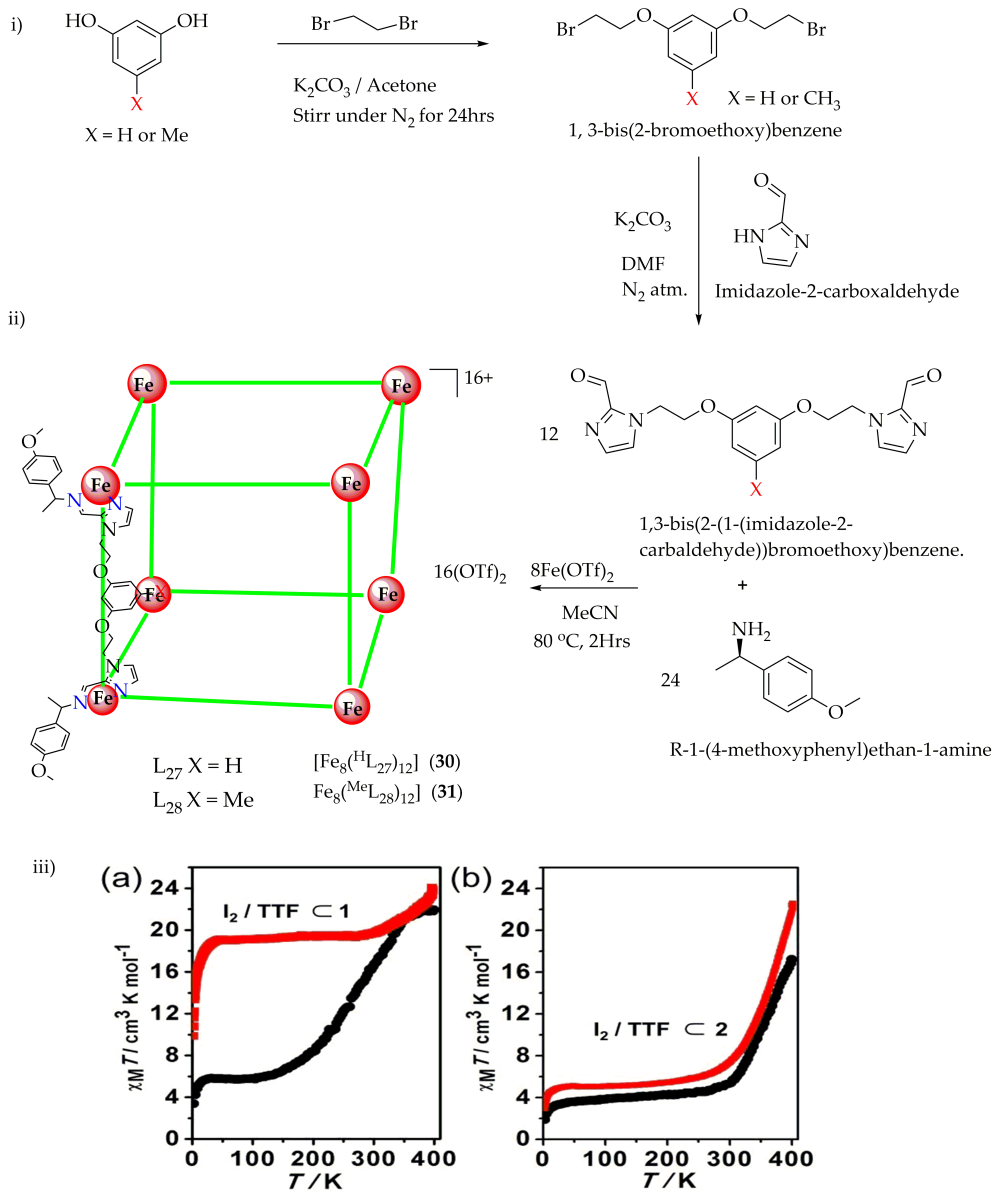
(**i**) Schematic representation for the synthesis of 1,3-bis(2-(1-(imidazole-2-carbaldehyde))bromoethoxy)benzene; (**ii**) schematic self-assembly of cubic cages of complexes **30** and **31;** (**iii**) χ_M_T vs. T of cubic cages of (**a**) [Fe_8_(^H^L_27_)_12_] black line and [I_2_/TTF@{Fe_8_(^H^L_27_)_12_}] red line (**b**) Fe_8_(^Me^L_28_)_12_] black line and [I_2_/TTF@{Fe_8_(^Me^L_27_)_12_}] red line. Reproduced with permission from [110]; Published by Royal Society of Chemistry, 2020.

Gu et al., reported on supramolecular complexes of [Fe_8_^II^L_(27–28)12_] cubic metal-organic cages with semi-rigid ligands of L_27_ and L_28_ with different substitutions, which further self-assembled to supramolecular assemblies with three different porous cavities used for the synergistic adsorption of I_2_ and tetrathiafulvalene (TTF), and in which their solid-state spin-crossover behaviors were influenced by the adsorbed guest molecules [110]. The semi-rigid di(imidazole aldehyde) components with resorcinol synthesized accordingly to give 1,3-bis(2-(1-(imidazole-2-carbaldehyde))bromoethoxy)benzene (Figure 19i). It was semi-rigid because the alkyl chains on both sides of the aromatic rings can enhance the flexibility of the ligands, while the benzene rings provided the rigidity. The self-assembly reactions of the di(imidazole aldehyde) components, R-1-(4-methoxyphenyl)ethan-1-amine and iron(II) trifluoromethanesulfonate in acetonitrile solution responsible for the formation of Fe^II^_8_L_12_ cubic cages of [Fe_8_(^H^L_27_)_12_] (**30**) and Fe_8_(^Me^L_28_)_12_] (**31**) (Figure 19ii).

The χ_M_T value for the supramolecular complex **30** (Figure 19iii(a)) is at 5.96 cm^3^ Kmol^−1^, showing 66%LS-24% HS from the temperature range of 2–150 K. As temperature range increases reaching 298 K (room temperature), the value increases gradually to 16.05 cm^3^Kmol^−1^ at which the spin state shows 23%LS-67%HS. At 400 K, the value reached a maximum of 22.08 cm^3^ K mol^−1^ indicating spin transitions to fully HS of 8Fe centers. Generally, the χ_M_T results of a supramolecular cubic **30** metal-organic cage has a gradual spin crossover behavior, and its transition temperature T_1/2_ is around 256 K, which is slightly lower than room temperature. However, the complex of **31** mostly remains at LS state (Figure 19iii(b)). The χ_M_T value increases slightly from 3.47 to 5.29 cm^3^ Kmol^−1^ as the temperature increases from 30 K to room temperature, indicating 78%Fe LS-22%Fe HS states. From room temperature to 400K, the value increases abruptly up to 17.38 cm^3^ K mol^−1^ and the spin states at this point shows 28%Fe LS-72% Fe HS centers. This generally shows abrupt and incomplete SCO behavior at 400 K.

Guest encapsulation affected the spin transitions of the two complexes. Encapsulating I_2_ and TTF molecules to complex **30,** i.e., [I_2_/TTF@{Fe_8_(^H^L_27_)_12_}] (Figure 19iii(a)) red, resulted in the stabilization of HS states of iron(II) centers. The same guests for complex **31** forming [I_2_/TTF@{Fe_8_(^Me^L_27_)_12_}] slightly affected the SCO (Figure 19iii(b) red), which is a very small change due to the radical cation of TTF, and also, complex **31** adsorbed less guests than complex **30**. Generally, guest molecules have a remarkable effect on spin transition nature [111,112,113].

## 4. Conclusions

The present review summarizes (Table 2) various classes of structurally characterized SCO-active multi-nuclear iron(II) Schiff base complexes in various dimensionalities. They are of various supramolecular arrays, such as spirals (helicate/meso-helicate), grids, cages and cubic. It has been concluded from the discussed articles that the spin state switching in the molecular architecture can be tuned by the careful design of ligands around the iron(II) centers, which in turn facilitates the communication between the metal centers and the ligands in getting the desired SCO properties. Also, the cooperativity in the metal centers facilitates different kinds of SCO behavior (gradual, abrupt, with hysteresis, two-step transition, incomplete, low spin or high spin). It has been understood from the present study that the Schiff bases act as effective ligands in attaining the desired architecture and tuning the ligand field of the aggregates. 

However, the attaining of the expected cooperative effects remains a big challenge, since the same architecture can display different SCO behaviors depending on the external environments, such as solvation, counter ions and host-guest encapsulations. Also, variation in the spin state of different metal centers in an aggregate at a particular temperature is an unexpected challenge facing the multi-nuclear systems discussed. In other words, even if in a structural point of view, it is interesting that from the magnetic point of view those deviate from the expected level. On comparing the SCO chemistry of mono-nuclear complexes to multi-nuclear systems reported, the mononuclear systems are straight forward and promising compared to the multi-nuclear systems with respect to SCO temperature, abruptness and hysteresis. Multi-nuclear systems still have to travel a lot to achieve this goal, and much more theoretical experimental investigations have to be invested to solve the challenges faced so far. 

In closing, the notable results reviewed herein for the past ten years from various research groups give insight into the very promising field of iron(II) multi-nuclear SCO architectures with Schiff base ligands, and they will also encourage the quest for further investigations with desired properties.

## Figures and Tables

**Table 1:** Summary of the Fe (2 × 2) grid spin states that read from the crystallographic data at 120 and 290 K for gradual deprotonations (Fe-8H→Fe-6H→Fe-4H).

	Fe-8H			Fe-6H			Fe-4H
	120 K	290 K	SCO of Individual Fe Centers	120 K	290 K	SCO of Individual Fe Centers	120 K
Fe1	LS	HS	SCO	LS	LS	No SCO	LS
Fe2	LS-HS	LS-HS	Disorder	LS-HS	HS	SCO	LS-HS
Fe3	LS	HS	SCO	LS	LS	No SCO	LS
Fe4	LS-HS	LS-HS	Disorder	LS-HS	HS	SCO	LS-HS
Nature of Spin state of the grid	Mixed	Mixed		Mixed	Mixed		Mixed

**Table 2:** Coordination environments and SCO properties of iron(II) supramolecular Schiff base complexes.

Supramolecular Complex and Number	Nuclearity	Coordination Environment	Coordination Structure	SCO Temperature (_T1/2_)	Ref.
[Fe_2_(H_2_L_1_)_3_]^4+^ (**1**)	Dinuclear	N_6_	Triple helicate	240 K	[57,59]
[Fe_2_(H_2_L_2_)_3_]^4+^ (**2**)	Dinuclear	N_6_	Triple helicate	-	[57,59]
[Fe_2_(L_3_)_3_] (**3**)	Dinuclear	N_6_	Meso-helicate	-	[67]
[Fe_2_(L_4_)_3_] (**4**)	Dinuclear	N_6_	Meso-helicate	400 K	[67]
[Fe_2_(L_5_)_3_](PF_6_)_4_ (**5**)	Dinuclear	N_6_	Triple helicate	165 K	[70]
[Fe_2_(L_5_)_3_](BF_4_)_4_ (**6**)	Dinuclear	N_6_	Triple helicate	-	[70]
[Fe_2_(L_5_)_3_](ClO_4_)_4_ (**7**)	Dinuclear	N_6_	Triple helicate	50 K	[70]
[Fe_2_(L_6_)_3_](ClO_4_)_4_ (**8**)	Dinuclear	N_6_	Triple helicate	140 K	[71]
[Fe_2_(H_2_L_8_)_3_](ClO_4_)_4_.1.5H_2_O (**9**)	Dinuclear	N_6_	Meso-helicate	212 K, 134 K	[72]
[Fe_2_(μ-*o*-NTrz)_3_(*o*-NTrz)_2_(NCS)_4_]·3MeOH (**10**·3MeOH)	Dinuclear	N_6_		135 K	[82]
[Fe_2_(μ-*o*-NTrz)_3_(*o*-NTrz)_2_(NCS)_4_]·3H_2_O (**10**.3H_2_O)	Dinuclear	N_6_		T_1/2↓↑_:150, 172 K	[82]
[Fe_2_(μ-*o*-NTrz)_3_(*o*-NTrz)_2_(NCS)_4_] (**10**·Ø)	Dinuclear	N_6_		175 K	[82]
[Fe_4_(L_11_)_4_](BF_4_)_8_ (**11**)	Tetranuclear	N_4_O_2_	Grid	-	[87]
[Fe_4_(L_13_)_4_]Cl_4_ (**12** ),	Tetranuclear	N_6_	Grid	400 K	[94]
[Fe_4_(L_13_)_4_](BF_4_)_4_ (**13**)	Tetranuclear	N_6_	Grid	400 K	[94]
[Fe_4_(L_13_)_4_](ClO_4_)_4_ (**14**)	Tetranuclear	N_6_	Grid	-	[94]
[Fe_4_(H_2_L_14_)_4_](BF_4_)_8_ (**15**)	Tetranuclear	N_6_	Grid	-	[95]
[Fe_4_(HL_14_)_2_(H_2_L_14_)_2_](BF_4_)_8_ (**16**)	Tetranuclear	N_6_	Grid	-	[95]
[Fe_4_(L_14_)_2_(H_2_L_14_)_2_](BF_4_)_8_ (**17**)	Tetranuclear	N_6_	Grid	-	[95]
[Fe_4_(L_15_)_4_](BF_4_)_8_ (**18**) fresh synthesis	Tetranuclear	N_6_	Tetrahedral cage	284 K	[96]
[Fe_4_(L_15_)_4_](BF_4_)_8_ (**18**) diluted with acetone	Tetranuclear	N_6_	Tetrahedral cage	288 K	[96]
[Fe_4_(L_15_)_4_](BF_4_)_8_ (**18**) dried	Tetranuclear	N_6_	Tetrahedral cage	-	[96]
[Fe_4_(L_16_)_4_](OTf)_8_ (**19**)	Tetranuclear	N_6_	Tetrahedral cage	-	[98]
[Fe_4_(L_17_)_4_](OTf)_8_ (**20**)	Tetranuclear	N_6_	Tetrahedral cage	-	[98]
[Fe_4_(L_17_)_4_](OTf)_8_ (**20**) Br^−^ encapsuled	Tetranuclear	N_6_	Tetrahedral cage	328 K	[98]
[Fe_4_(L_17_)_4_](OTf)_8_ (**20**) CS_2_ encapsuled	Tetranuclear	N_6_	Tetrahedral cage	324 K	[98]
[Fe_4_(L_18_)_4_](BF_4_)_8_ (**21**) (28.16CH_3_CN)-solvated	Tetranuclear	N_6_	Tetrahedral cage	-	[99]
[Fe_4_(L_18_)_4_](BF_4_)_8_ (**21**)-non solvated	Tetranuclear	N_6_	Tetrahedral cage	-	[99]
[Fe_4_(L_19_)_6_]^8+^ (**22**)	Tetranuclear	N_6_	Tetrahedral cage	-	[48]
[Fe_4_(L_20_)_6_]^8+^ (**23**)	Tetranuclear	N_6_	Tetrahedral cage	-	[48]
[Fe_4_(L_21_)_6_]^8+^ (**24**)	Tetranuclear	N_6_	Tetrahedral cage	-	[48]
[Fe_8_(PtL_22_)_6_]^28+^ (**25**)	Octanuclear	N_6_	Cubic	215 K	[104]
[Fe_8_(PtL_23_)_6_]^28+^ (**26**)	Octanuclear	N_6_	Cubic	281 K	[104]
[Fe_8_(PtL_24_)_6_]^28+^ (**27**)	Octanuclear	N_6_	Cubic	324 K	[104]
[Fe_8_L_25_]^16+^ (**28**)	Octanuclear	N_6_	Cubic	249.6 K	[105]
[Fe_8_L_25_]^16+^ (**28**) (C_70_ encapsuled)	Octanuclear	N_6_	Cubic	229.9 K	[105]
[Fe_8_L_26_]^16+^ (**29**)	Octanuclear	N_6_	Cubic	247.7 K	[105]
[Fe_8_L_26_]^16+^ (**29**) (C_70_ encapsuled)	Octanuclear	N_6_	Cubic	236.3 K	[105]
[Fe_8_(^H^L_27_)_12_] (**30**)	Octanuclear	N_6_	Cubic	256 K	[110]
[Fe_8_(^Me^L_28_)_12_] (**31**)	Octanuclear	N_6_	Cubic	-	[110]

## Data Availability

Not applicable.
